# Dynamic changes of rumen microbiota and serum metabolome revealed increases in meat quality and growth performances of sheep fed bio-fermented rice straw

**DOI:** 10.1186/s40104-023-00983-5

**Published:** 2024-02-28

**Authors:** Yin Yin Kyawt, Min Aung, Yao Xu, Zhanying Sun, Yaqi Zhou, Weiyun Zhu, Varijakshapanicker Padmakumar, Zhankun Tan, Yanfen Cheng

**Affiliations:** 1 Laboratory of Gastrointestinal Microbiology, National Center for International Research on Animal Gut Nutrition, Nanjing Agricultural University, Nanjing, 210095 China; 2 Department of Animal Nutrition, University of Veterinary Science, Nay Pyi Taw 15013, Myanmar; 3 International Livestock Research Institute, Nairobi, 00100 Kenya; 4 College of Animal Science, Tibet Academy of Agricultural and Animal Husbandry Sciences, Lhasa, 850000 China; 5 State Key Laboratory of Grassland Agro-Ecosystems, Center for Grassland Microbiome, College of Pastoral Agriculture Science and Technology, Lanzhou University, Lanzhou, 730000 China

**Keywords:** Bio-fermentation, Growth rate, Meat quality, Metabolome, Microbiota, Rice straw

## Abstract

**Background:**

Providing high-quality roughage is crucial for improvement of ruminant production because it is an essential component of their feed. Our previous study showed that feeding bio-fermented rice straw (BF) improved the feed intake and weight gain of sheep. However, it remains unclear why feeding BF to sheep increased their feed intake and weight gain. Therefore, the purposes of this research were to investigate how the rumen microbiota and serum metabolome are dynamically changing after feeding BF, as well as how their changes influence the feed intake, digestibility, nutrient transport, meat quality and growth performances of sheep. Twelve growing Hu sheep were allocated into 3 groups: alfalfa hay fed group (AH: positive control), rice straw fed group (RS: negative control) and BF fed group (BF: treatment). Samples of rumen content, blood, rumen epithelium, muscle, feed offered and refusals were collected for the subsequent analysis.

**Results:**

Feeding BF changed the microbial community and rumen fermentation, particularly increasing (*P* < 0.05) relative abundance of *Prevotella* and propionate production, and decreasing (*P* < 0.05) enteric methane yield. The histomorphology (height, width, area and thickness) of rumen papillae and gene expression for carbohydrate transport (*MCT1*), tight junction (claudin-1, claudin-4), and cell proliferation (*CDK4*, *Cyclin A2*, *Cyclin E1*) were improved (*P* < 0.05) in sheep fed BF. Additionally, serum metabolome was also dynamically changed, which led to up-regulating (*P* < 0.05) the primary bile acid biosynthesis and biosynthesis of unsaturated fatty acid in sheep fed BF. As a result, the higher (*P* < 0.05) feed intake, digestibility, growth rate, feed efficiency, meat quality and mono-unsaturated fatty acid concentration in muscle, and the lower (*P* < 0.05) feed cost per kg of live weight were achieved by feeding BF.

**Conclusions:**

Feeding BF improved the growth performances and meat quality of sheep and reduced their feed cost. Therefore, bio-fermentation of rice straw could be an innovative way for improving ruminant production with minimizing production costs.

**Graphical Abstract:**

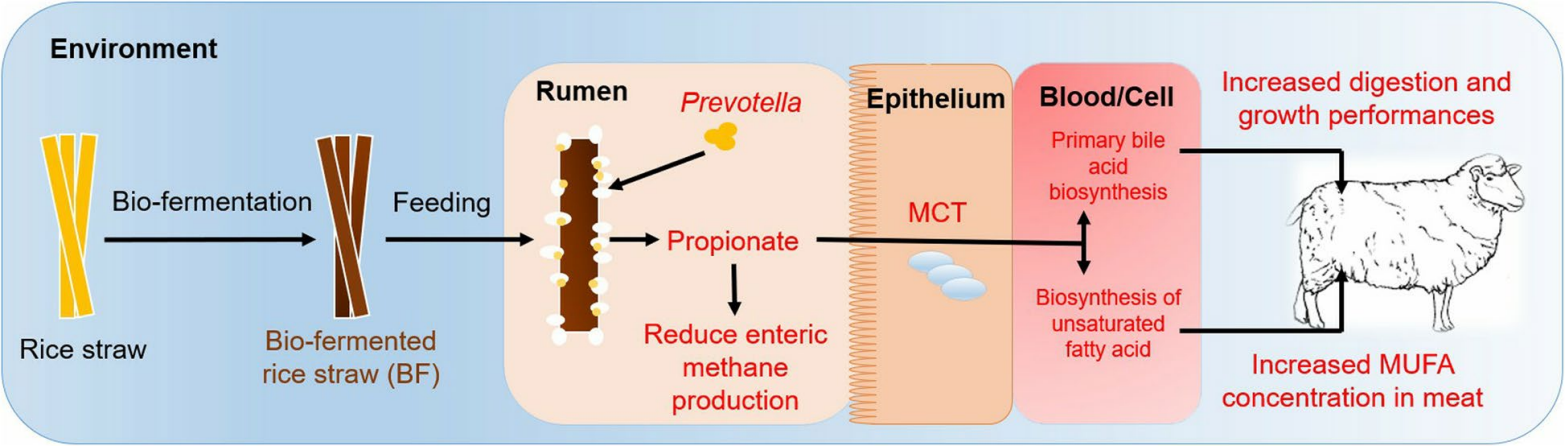

**Supplementary Information:**

The online version contains supplementary material available at 10.1186/s40104-023-00983-5.

## Introduction

Global ruminant production is challenged in recent years due to increasing demand for meat and milk. For improvement of ruminant production, providing high-quality roughage is critical because it is an essential component of ruminant feed. Alfalfa hay (AH) has been accepted as a high-quality roughage because of its excellent protein and mineral contents [1]. With the rapid expansion of the animal husbandry industry in China, it has shown an increasing demand for alfalfa in recent years. However, China produces comparatively less alfalfa, with the average self-sufficiency rate of 64% [2], requiring imports to meet demand. China’s import of AH increased from 0.44 million tons to 1.36 million tons between 2012 and 2020 [3]. Although AH is a high-quality roughage, its price is quite costly, which increases the input cost for ruminant production. Therefore, it's critical to take into consideration of potentially high-quality alternate roughage and agricultural by-products to replace AH. Rice straw (RS), as a potential source of roughage for ruminants, has the advantages of low cost and abundant availability, but its cellulose-hemicellulose-lignin complex limits its use by microbes and enzymes in the rumen, resulting in low ruminal degradation and animal performances [4]. Several methods, such as chemical, physical and biological, have been successfully developed to improve the degradability of high fiber forages and the efficiency of fermentation in the rumen [5]; however, they have limitations. Chemical methods frequently pollute the environment, whereas physical methods are costly due to high energy requirements [6]. Biological methods have become recognized as low-energy demanding and environmentally friendly, because they produce no effluent during the process [7].

Bio-fermentation is a biological process that involves combining beneficial microorganism strains into a multi-strain complex and then inoculating it into a substrate to releases soluble sugars from lignocellulose for utilization by rumen microorganisms. This process increases rumen cellulose and hemicellulose degradation, thus improving nutritional value [8]. Lactic acid bacteria (LAB) inoculation could improve silage quality and feed efficiency of low-quality forage such as RS [9], reduce fiber content and increase dry matter and fiber digestibility [10], and enhance the microbial community in the rumen [11].

The rumen microbiota is responsible for cellulose degradation by colonization of ingested roughage and excretion of fiber-degrading enzymes [12]. Dietary composition influences the structure and metabolic activity of the rumen microbial community [13]. Interestingly, the roughage sources have been commonly recognized as a potential target for manipulation to regulate ruminal microbiota metabolism and increase the growth performance of animals [14]. Within the rumen, the dynamic changes of bacterial colonization and gene function of microbiota associated with RS and AH differ [15]. Thus, dynamic changes in the microbial community could contribute to understanding how foraging and ruminal microbes interact [16]. In addition, serum metabolites are an important tool for assessing the impact of nutrition on animal health and metabolism. Thus, metabolomics could provide information regarding animal metabolite profiles and integrated metabolic pathways in response to nutritional intervention [17]. Changes in serum metabolome may reflect the effects of nutritional interventions on energy and nutrient metabolism; some of these metabolites have been identified as being directly related to animal performance and meat quality [18].

Our previous studies revealed that bio-fermentation altered the physical structure and nutritional qualities of RS [19], as well as in vitro rumen fermentation and the tightly attached bacteria [20]. Sheep fed bio-fermented rice straw (BF) had higher feed intake and average daily gain (ADG) than sheep fed RS [19]. Thus, BF has a potential to relieve much of feed shortage in large areas of China. However, it remains unclear why feeding BF to sheep increased their feed intake and weight gain. It was hypothesized that the feeding of BF would alter the fermentation products, rumen microbiota, and serum metabolome, ultimately leading to improved growth performances in the sheep. Therefore, the purposes of this research were to investigate how the rumen microbiota and serum metabolome are dynamically changing after feeding BF, as well as how their changes influence the feed intake, digestibility, nutrient transport, meat quality and growth performances of sheep.

## Materials and methods

### Preparation of experimental feeds

The AH, RS and BF used in this experiment were purchased from Zhongxin Agricultural Service Professional Cooperative, Yancheng City, Jiangsu Province, China. For bio-fermentation, RS was picked up after harvesting rice in the field and shipped to the factory. The “S102 straw micro-storage” silage inoculant was supplied by the Jiangsu Academy of Agricultural Sciences. The rate of application of inoculant was 2 × 10^8^ CFU/kg RS. Thereafter, it was wrapped in a polyethylene sheet and fermented for 42 d.

### Experimental animals, feeds and management

Twelve three-month-old male Hu sheep with fistulas, weighing 25 ± 3.02 kg, were confined in individual pens (1.2 m × 1.4 m) with a feed manger and an automatic drinker. The experiment lasted for 50 d with 21 d for adaptation and 29 d for formal trial. During adaptation period, all experimental sheep were offered the total mixed ration (40% AH and 60% concentrate as the dry matter basis). They were then allocated into 3 groups according to the complete randomized design: AH fed group (AH: positive control), RS fed group (RS: negative control) and BF fed group (BF: treatment), with 4 replicates and fed their respective feed during formal trial. Experimental diets were formulated according to the guideline of Chinese sheep nutrient requirement (Table 1, Additional file 1: Table S1). The sheep were fed twice daily at 08:00 and 16:00, allowing up to 10% refusal and free access to drinking water. On 29 d of formal trial, the sheep were weighed and then slaughtered by professional abattoir personnel in accordance with animal welfare regulations for slaughter.

**Table 1:** Ingredient compositions and nutritive values of experimental feeds (dry matter basis)

**Items**	**AH**	**RS**	**BF**
Ingredient, %			
Alfalfa hay	40.00	0	0
Rice straw	0	40.00	0
Bio-fermented rice straw	0	0	40.00
Corn	30.00	33.00	33.00
Bean pulp	0	18.50	18.50
Wheat middling	24.50	5.00	5.00
Zeolite	2.50	0	0
Limestone	0	0.50	0.50
Premix	2.50	2.50	2.50
NaCO_3_	0.50	0.50	0.50
Nutritive values			
DE, kcal/kg	2,880	2,910	2,926
ME, kcal/kg	2,908	2,938	2,955
DM, %	83.21	82.33	67.85
CP, %	12.80	12.48	12.57
EE, %	2.66	2.29	2.20
Ash, %	12.00	10.30	10.32
NDF, %	26.65	33.69	31.63
ADF, %	12.16	15.48	15.25
ADL, %	1.25	1.83	1.36
Ca, %	1.04	0.97	0.99
TP, %	0.42	0.42	0.42

*AH* Alfalfa hay, *RS* Rice straw, *BF* Bio-fermented rice straw, *NaCO*_*3*_ Sodium bicarbonate, *DE* Digestible energy, *ME* Metabolizable energy, *DM* Dry matter, *CP* Crude protein, *EE* Ether extract, *NDF* Neutral detergent fiber, *ADF* Acid detergent fiber, *ADL* Acid detergent lignin, *Ca* Calcium, *TP* Total phosphorus

### Determination of growth performances

Daily feed offered, refusal and weekly body weight were recorded to calculate average daily feed intake, total weight gain, ADG, feed efficiency and feed cost effectiveness. The feed refusal of each sheep was recorded and removed before morning feeding. The daily feed intake was calculated with the equation: feed intake = feed offered – feed refusal. Body weight was also measured before morning feeding of scheduled days. The equations used for calculation of growth rate were: total weight gain = final body weight – initial body weight, and ADG = total weight gain/day of experiment period. The feed efficiency was calculated with the equation: feed efficiency = ADG/daily feed intake. Feed cost analysis was based on the actual cost for daily feed intake and weight gain. The equations used for feed cost effectiveness were: total feed cost = total feed intake × unit price, and feed cost per kg of live weight gain = total feed cost/total weight gain. The digestion trial was conducted at the last 5 days of the experiment to calculate the digestibility. For the determination of feed digestibility, acid insoluble ash (AIA) was used as an internal marker, and digestibility was calculated according to the model: nutrient digestibility = 100 – 100 × (% indicator in feed × % nutrient in feces)/(% indicator in feces × % nutrient in feed) [21]. Dry matter (DM), organic matter (OM), crude protein (CP) and AIA contents were analyzed according to AOAC [22], and the content of neutral detergent fiber (NDF) and acid detergent fiber (ADF) were analyzed by the ANKOM filter bag technique using an ANKOM 200i fiber analyzer (ANKOM Technologies, Inc., Fairport, New York, USA).

### Determination of the meat quality

After slaughtering on 29 d, *longissimus dorsi* (LD) muscle were collected between the 9^th^ and 13^th^ ribs from the right side of the carcasses, of which one was stored at 4 °C for subsequent physical analysis, and the other one was stored at −20 °C for intramuscular fat and muscle fatty acid analysis. The pH of muscle was measured at 24 h after slaughter by portable pH meter (Testo Instrument Co., Ltd., Lenzkirch, Germany). The L* (lightness), a* (redness), and b* (yellowness) of the LD muscle were recorded 24 h after slaughter by Minolta CR-10 colorimeter (Konica Minolta Inc., Osaka, Japan). The dripping and cooking losses were analyzed according to the report [23]. Warner-Bratzler shear force (WBSF) was tested with a digital tenderness meter (C-LM3B, Tenovo, Beijing, China) [24]. Fat content in muscle was analyzed with the procedures of AOAC [21]. The fatty acid composition was measured by fatty acid methyl ester synthesis [25], whereas fatty acid was extracted, and then the Agilent high-performance gas chromatograph was used for the measurement.

### Determination of serum metabolome

Blood samples were collected from jugular vein using vacutainer tubes, before morning feeding on 1, 7, 14, 21, and 28 d of experiment. Then, they were centrifuged at 3,000 × *g* for 20 min and serum was stored at −20 °C for analysis of blood biochemical indicators, and −80 °C for determination of blood metabolite. The concentrations of serum biochemical indices, including total protein (TP), albumin (ALB), globulin (GLB), glucose (GLU), urea (UREA), total cholesterol (TCHO), triglycerides (TRIG), high-density lipoprotein (HDL), alanine aminotransferase (ALT), aspartate aminotransferase (AST) and alkaline phosphatase (ALP) were measured by an automatic biochemical analyzer (SRL, Inc., Tokyo, Japan). The serum metabolome was analyzed by liquid chromatography-mass spectrometry (LC-MS).

#### Liquid chromatography-mass spectrometry (LC-MS) analysis

One hundred μL of sample and 300 μL of methanol (Merck, Darmstadt, Germany) were added in a 1.5-mL centrifuge tube and vortexed for 30 s to mix. The tube was stand at −40 °C for 1 h, and vortexed for 30 s. Then, it stood at 4 °C for 0.1 h and centrifuged for 15 min at 12,000 r/min and 4 °C. All the supernatant in the centrifuge tube was taken and stood at −40 °C for 1 h, then centrifuged for 15 min at 12,000 r/min and 4 °C again. Two hundred μL of supernatant and 5 μL of 1 mg/mL DL-o-chlorophenylalanine (internal standard; GL-Biochem Ltd., Shanghai, China) were transferred to the injection vial. Ten μL of serum samples were injected into the LC-MS system (Waters, UPLC; Thermo, Q Exactive) with Waters XBridge Amide column (4.6 mm × 150mm, 3.5 μm) and maintained at 40 °C and flow rate of 0.3 mL/min. Parameters for positive ion mode were as follows: Heater temperature 300 °C, sheath gas flow rate 45 arb, aux gas flow rate 15 arb, sweep gas flow rate 1 arb, spray voltage 3.0 kV, capillary temperature 350 °C, S-Lens RF Level 30%. Parameters for negative ion mode were as follows: Heater temperature 300 °C, sheath gas flow rate 45 arb, aux gas flow rate 15 arb, sweep gas flow rate 1 arb, spray voltage 3.2 kV, capillary temperature 350 °C, S-Lens RF Level 60%.

### Determination on histomorphology and gene expression of rumen epithelium

The rumen was taken out immediately after slaughtering and the empty rumen was then sampled. A piece of rumen epithelial tissue was cut to 5 cm × 5 cm for the determination of rumen epithelial papilla-related indicators. Another piece of rumen epithelial tissue with a thickness of about 8 μm was cut and fixed in 4% paraformaldehyde solution, made into paraffin sections and stained, and the structure of the rumen papilla was measured with an optical microscope. Another rumen epithelial tissue was separated from the muscle layer and rinsed with PBS. These mucosal samples were cut into pieces and put into cryopreservation tubes, and immediately transferred to a liquid nitrogen tank for storage, for RNA extraction and determination of related nutrient transport genes. The rumen epithelial tissue, and their length and width were measured by using a vernier caliper. The observation of histomorphology was carried out according to the blind inspection method [26].

### Rumen epithelial RNA extraction and fluorescent quantitative PCR

Rumen epithelial samples were ground into powder and the ultra-pure total RNA rapid extraction kit was used to extract the total RNA of the rumen epithelium. An ultra-micro spectrophotometer was used to measure the concentration and purity of the extracted RNA [27]. The 1.4% agarose gel electrophoresis was also used to check RNA integrity. The 1 μg of qualified RNA samples were immediately reverse transcribed into cDNA using a reverse transcription kit and stored in a −20 °C refrigerator. Quantification of gene expression for nutrient transport was determined using commercially synthesized primers (Additional file 2: Table S2; Sangon Bioengineering Co., Ltd., Shanghai, China). Quantitative real-time PCR analysis was performed using fluorescent quantitative QuantStudio^TM^ 5 Flex System and SYBR^®^ Premix Ex Tag kit. The 20 μL of reaction system premix included the SYBR GREEN 10 μL, ROXII 0.4 μL, forward and reverse primers 0.4 μL each, DNA template 2 μL, enzyme-free water 6.8 μL. Then, 18 μL of reaction system premix was add to each well of PCR plate, and then add 2 μL of DNA template, seal the plate and centrifuge at 3,000 r/min for 1 min, react on the machine. The fluorescence reaction program was: 95 °C for 30 s; 95 °C for 5 s, 60 °C for 30 s, 40 cycles; 95 °C for 15 s, 60 °C for 1 min, and 95 °C for 15 s. Each sample contained replicates of 3 wells, and each batch of assays contained a negative control and a negative blank. Finally, with the expression of the internal reference gene *GAPDH* as a reference, the relative expression of the target gene was calculated using the 2^-∆∆CT^ method.

### Determination of rumen fermentation products and microbial community

Rumen content samples were collected before morning feeding on 1, 2, 3, 4, 5, 6, 7, 14, 21, and 28 d of experiment and stored in liquid nitrogen tank for the determination of rumen metabolites and the extraction of rumen microbial DNA.

#### Analysis of rumen fermentation products and estimation of methane yield

The pH of rumen content was measured with a pH meter (Ecoscan pH 5, Singapore). Lactate was determined with the Lactate Assay kit (Nanjing Jiancheng Bioengineering Institute, Nanjing, Jiangsu, China) and ammonia nitrogen (NH_3_-N) was measured with the method of Weatherburn [28]. Microbial protein (MCP) was determined with Bradford Protein Assay kit (Beijing Solarbio Science and Technology, Beijing, China). A gas chromatograph (GC-2014AFsc, Shimadzu, Kyoto, Japan) was used for determination of volatile fatty acids (VFAs) with the following conditions: column temperature of 135 °C, injection temperature of 200 °C, flame ionization detector temperature of 200 °C, and carrier gas (N_2_) pressure of 0.06 MPa.

Methane yield (g) per kg of dry matter intake (DMI) was estimated by the model [29]: MY = s/P + t, where MY means methane yield, P means propionate, s means constant, and t means coefficient.

#### Analysis of rumen bacterial community by Illumina Hiseq sequencing

The 0.3 g of rumen content samples were used for DNA extraction using the bead-beating and phenol–chloroform extraction method [30]. After DNA extraction, a PCR thermal cycler (Eppendorf AG 22331, Hamburg, Germany) was used to amplify the total bacterial 16S rRNA gene. The universal primers, 515F 5′-GTGCCAGCMGCCGCGGTAA-3′ and 806R 5′-GGACTACHVGGGTWTCTAAT-3′ [31], targeting the 16S rRNA gene were used to obtain the PCR amplicons of total bacteria. The PCR amplicons were then purified by means of Agencourt AMPure XP beads (Beckman Coulter, Milan, Italy). The RNA concentration was quantified with a Small RNA kit (Agilent Technologies, 5067-1548, Beijing, China) and 2100 Bioanalyzer. Amplified libraries were sequenced on an Illumina Hiseq platform at BGI Life Tech Co., Ltd. (Beijing, China).

To remove ambiguous and low-quality sequences, the raw sequencing data were preprocessed with cut adapt v2.6 software [32]. After trimming, the sequence data were further quality-filtered to abandon reads with ambiguous, homologous sequences. If the window average quality value was < 20, the end of the read sequence was truncated from the window, and the reads with a final read length < 75% of the original read length were removed. Then, the reads with chimera were detected and removed by QIIME 2 software [33]. After the pretreatment described above, clean reads were grouped into amplicon sequence variant (ASV) using V search software at a 99% similarity level. The representative read of each ASV was selected by using the QIIME in bacterial community and was annotated by the SILVA 16S rRNA database. Alpha diversity, as indicated by the number of ASV, Evenness, Faith’s phylogenetic diversity (Faith_pd), and Shannon, was calculated with QIIME 2 software. Evenness described the relative abundance of the different species making up the richness. Faith_pd was used to calculate the alpha diversity. The Shannon index was used for microbial diversity analysis. A Venn diagram was used to visualize the number of common and unique features. The unweighted UniFrac distance was used for principal coordinate analysis (PCoA) to compare the microbial communities between two groups. Linear discriminant analysis effect size (LEfSe) analysis was also employed to determine the significant differences in the bacterial community between the two groups. Tax4Fun analysis was performed to predict the functional capabilities of microbial communities based on 16S data.

### Data processing and analysis

Venn diagrams, PCoA analysis and Spearman’s correlation analysis were completed by the online data visualization and analysis tool (https://www.bioincloud.tech/task-meta/). The principal component analysis (PCA) and partial least squares-discriminate analysis (PLS-DA) were carried out in SIMCA-P software (Version 13, Umetrics AB, Sweden). Differentially expressed metabolites (DEMs) were identified according to variable importance in projection (VIP) > 1 and adjusted *P* < 0.05, which were obtained from PLS-DA and statistical analysis, respectively. Differential metabolite data were used for pathway analysis on the MetaboAnalyst 3.0 (http://www.metaboanalyst.ca). LEfSe analysis was performed by the online LEfSe analysis tool (http://huttenhower.sph.harvard.edu/galaxy/). Tax4Fun analysis was also performed by online tax4Fun (http://tax4fun.gobics.de/).

Data on the growth performances, cost effectiveness and meat qualities were analyzed using a one-way analysis procedure with Tukey's tests as post hoc. Serum biochemical indices and rumen fermentation parameters were analyzed by a two-way ANOVA using the General Linear Model procedure to determine the main effects, treatments and sampling times, and their interaction. SPSS (version 25.0, Chicago, IL, USA) was used for all statistical procedures. The probability values (*P* value) with a significance level of less than 0.05 were considered significant and were displayed in the corresponding tables and figures. All data are presented as mean ± standard error of mean (SEM) and plotted in GraphPad Prism 8.0.

## Results

### Growth performances and meat quality

Sheep fed AH and BF showed higher (*P* < 0.05) dry matter intake, digestibility and ADG compared to sheep fed RS, resulting higher (*P* < 0.05) feed efficiency. Feed cost per kilogram of live weight gain was lowest (*P* < 0.05) in BF group, then followed by RS and AH group (Table 2). The pH, WBSF and meat color did not differ (*P* > 0.05) among the groups. The intramuscular fat content of RS and BF groups was lower (*P* < 0.05) than that of AH group. The lowest dripping and cooking losses were observed in BF group, followed by AH and RS groups. Saturated fatty acids (SFA) such as C18:0 and C20:0 were higher (*P* < 0.05) in RS group than in AH and BF groups, whereas C8:0, C13:0, C16:0 and C17:0 did not differ (*P* > 0.05). Monounsaturated fatty acid (MUFA) such as C18:1 *cis*-9 and C20:1 *cis*-11 of AH and BF groups were greater (*P* < 0.05) than those of RS group, while C14:1 *cis*-9, C16:1 *cis*-9 and C17:1 *cis*-10 were not different (*P* > 0.05). Polyunsaturated fatty acid (PUFA) such as C18:2n-6, C20:5n-3 and C22:6n-3 did not differ (*P* > 0.05), however C20:4n-6 of AH and BF groups was higher (*P* < 0.05) than those of RS group. Thus, the lower (*P* < 0.05) total SFA and higher (*P* < 0.05) total MUFA (ΣMUFA) concentrations were observed in AH and BF groups than RS group, whereas total PUFA (ΣPUFA) concentration did not differ (*P* > 0.05) among the groups. The ΣMUFA/ΣSFA ratio was higher (*P* < 0.05) in AH and BF groups than in RS group, while Σn-6 PUFA, Σn-3 PUFA and Σn-6/Σn-3 PUFA were not different (*P* > 0.05, Table 3).

**Table 2:** Effect of feeding BF on growth performances and feed cost effectiveness of sheep

Items	AH	RS	BF	SEM	*P*-value
Feed intake					
Total feed intake, kg	32.14^a^	25.50^b^	31.00^a^	1.18	0.028
Daily feed intake, kg/d	1.15^a^	0.91^b^	1.11^a^	0.04	0.028
Digestibility					
DM, %	73.70^a^	64.75^b^	71.78^a^	70.07	< 0.001
NDF, %	53.96^a^	46.29^b^	55.70^a^	51.98	< 0.001
ADF, %	43.64^ab^	40.66^b^	46.45^a^	43.58	0.023
Body weight gain					
Initial weight, kg	26.98	25.80	26.25	0.87	0.880
Final weight, kg	33.61	29.95	32.68	1.02	0.342
Total weight gain, kg	6.64^a^	4.15^b^	6.43^a^	0.36	< 0.001
Daily weight gain, kg/d	0.24^a^	0.15^b^	0.23^a^	0.01	< 0.001
Feed efficiency	0.21^a^	0.16^b^	0.21^a^	0.01	0.023
Cost effectiveness					
Cost for forage, RMB/d	1.84^a^	0.19^c^	0.32^b^	0.23	< 0.001
Cost for concentrate, RMB/d	1.94^ab^	1.84^b^	2.23^a^	0.08	0.073
Total cost, RMB/d	3.78^a^	2.02^c^	2.54^b^	0.23	< 0.001
Feed cost, RMB/kg live weight gain	15.98^a^	13.77^b^	11.09^c^	0.72	0.004

*AH* Alfalfa hay, *RS* Rice straw, *BF* Bio-fermented rice straw, *DM* Dry matter intake, *NDF* Neutral detergent fiber, *ADF* Acid detergent fiber

^a–c^Means within a row with different superscripts significantly different (*P* < 0.05)

**Table 3:** Effect of feeding BF on meat quality and fatty acid compositions of sheep

Items	AH	RS	BF	SEM	*P*-value
Meat quality					
pH_24h_	5.25	5.19	5.30	0.04	0.502
IMF^1^, %	11.70^a^	9.54^b^	9.47^b^	0.41	0.023
Dripping loss, %	8.07^b^	8.96^a^	7.00^c^	0.27	0.001
Cooking loss, %	23.82^a^	25.39^a^	18.37^b^	1.06	0.003
WBSF, N	55.78	57.36	56.38	2.62	0.975
Meat color_24h_					
L* (Brightness)	32.77	34.99	36.52	0.92	0.270
a* (Redness)	8.85	9.21	8.50	0.17	0.279
b* (Yellowness)	2.68	3.36	2.83	0.32	0.699
Meat fatty acid composition, % of total fatty acids					
Saturated fatty acid (SFA)					
C8:0	1.21	1.40	1.22	0.05	0.248
C13:0	3.44	4.17	3.59	0.15	0.097
C16:0	17.37	18.33	16.61	0.41	0.230
C17:0	3.49	4.19	3.58	0.14	0.063
C18:0	13.61^b^	15.74^a^	13.28^b^	0.45	0.032
C22:0	2.11^b^	3.72^a^	3.41^a^	0.29	0.039
Monounsaturated fatty acid (MUFA)					
C14:1 *cis*-9	8.69	9.95	8.83	0.51	0.586
C16:1 *cis*-9	0.89	0.10	0.89	0.02	0.133
C17:1 *cis*-10	1.11	1.07	1.08	0.01	0.519
C18:1 *cis*-9	18.68^a^	12.88^b^	18.62^a^	0.96	0.003
C20:1 *cis*-11	1.19^a^	0.82^b^	1.02^ab^	0.06	0.014
Polyunsaturated fatty acid (PUFA)					
C18:2n-6	13.10	12.83	12.94	0.13	0.731
C20:4n-6	11.47^a^	10.33^b^	11.40^a^	0.21	0.026
C20:5n-3	1.18	1.23	1.18	0.02	0.487
C22:6n-3	2.47	2.36	2.34	0.06	0.737
ΣSFA	41.23^b^	47.54^a^	41.69^b^	0.92	< 0.00
ΣMUFA	30.57^a^	25.72^b^	30.45^a^	0.74	< 0.001
ΣPUFA	28.20	26.75	27.86	0.31	0.125
ΣMUFA/ΣSFA	0.74^a^	0.54^b^	0.73^a^	0.03	< 0.001
Σn-6 PUFA	24.56	23.16	24.35	0.29	0.099
Σn-3 PUFA	3.64	3.59	3.52	0.06	0.750
Σn-6/Σn-3 PUFA	6.76	6.48	6.94	0.13	0.401

*AH* Alfalfa hay, *RS* Rice straw, *BF* Bio-fermented rice straw, *IMF* Intramuscular fat, *WBSF* Warner-Bratzler shear force, *ΣSFA* Total saturated fatty acid, *ΣMUFA* Total mono-unsaturated fatty acid, *ΣPUFA* Total poly-unsaturated fatty acid

^1^IMF was based on DM basis

^a–c^Means within a row with different superscripts significantly different (*P* < 0.05)

### Dynamic changes of serum metabolome

**Fig. 1:**
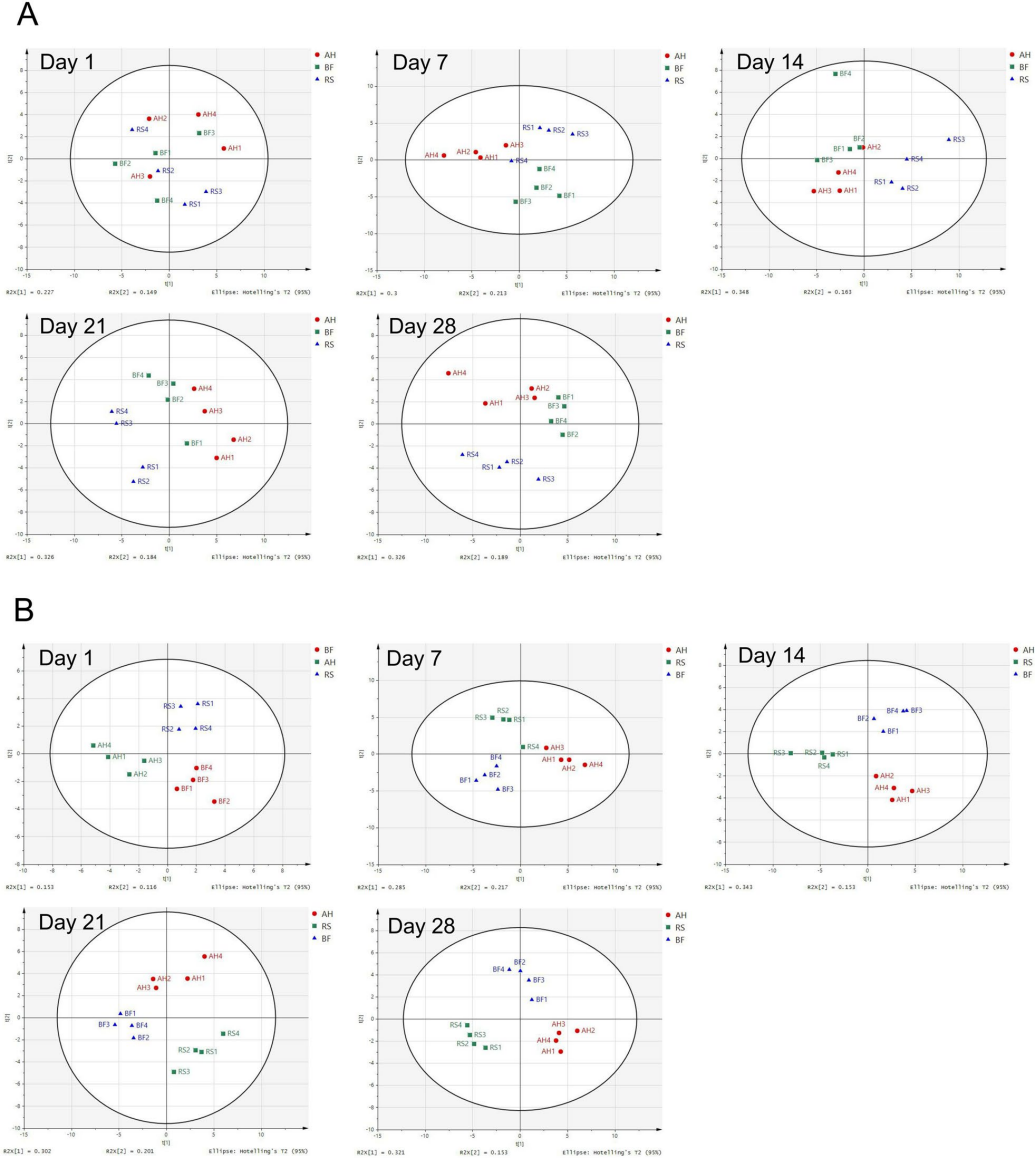
Dynamic changes of serum metabolome among AH, RS and BF groups. **A** Principal component analysis (PCA); **B** Partial least squares-discriminant analysis (PLS-DA)

**Fig. 2:**
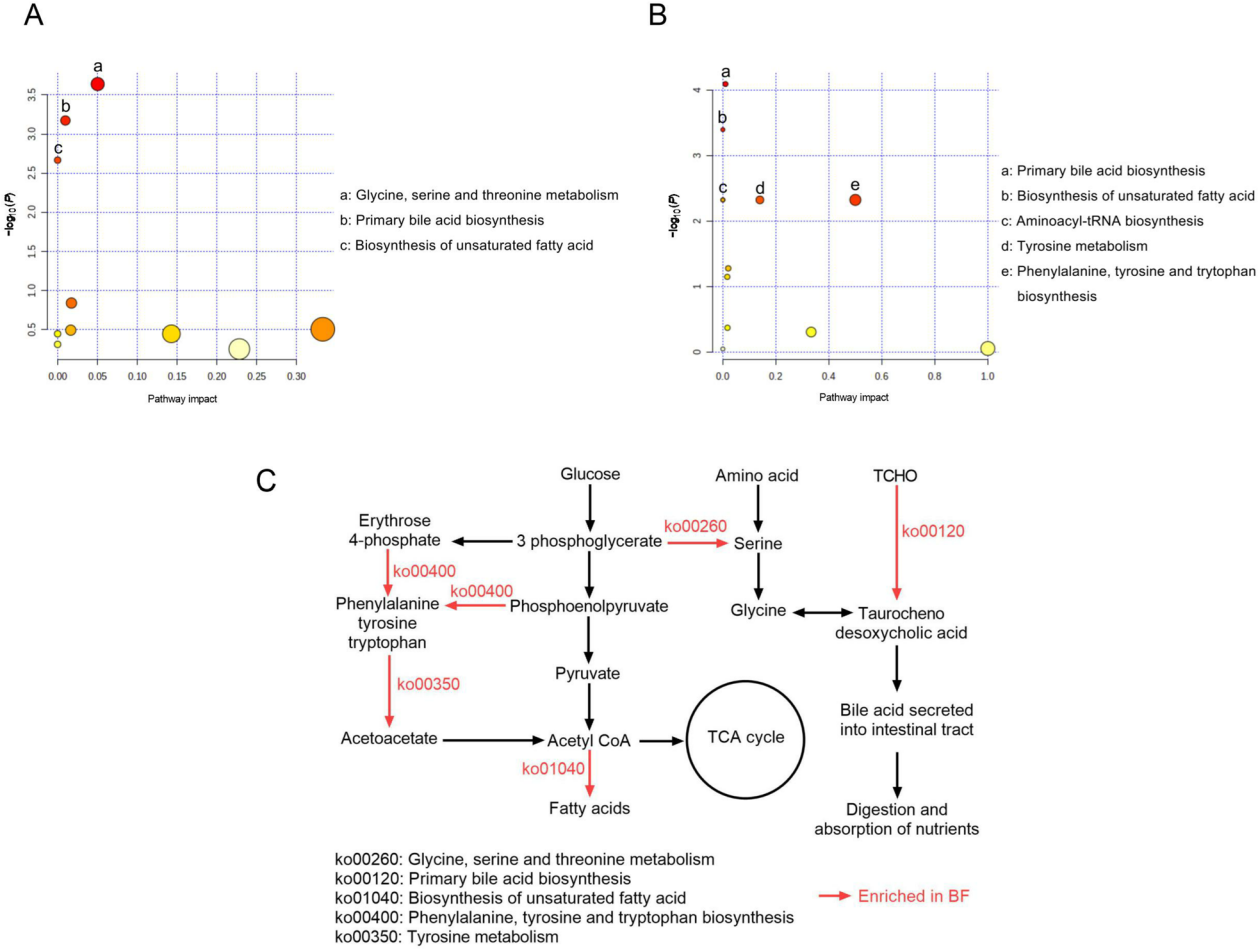
Metabolomic analysis among AH, RS and BF groups. **A** Pathway analysis between BF and AH groups; **B** Pathway analysis between BF and RS groups; **C** Metabolic pathways involved in biosynthesis of fatty acid and bile acid

The concentrations of serum AST, total protein, albumin, and glucose were higher (*P* < 0.05) in AH and BF groups than in RS group (Additional file 3: Table S3). A total of 368 metabolites were detected, of which 53 (relative abundance > 1,000, based on 1,000,000) were used for the analysis of PCA and PLS-DA. The PCA score plots showed dynamic changes of serum metabolome and revealed that the first and second PCs explained 22.7% and 14.9%, 30.0% and 21.3%, 34.8% and 16.3%, 32.6% and 18.4%, and 32.6% and 18.9% of the variations of 1, 7, 14, 21 and 28 d, respectively. According to the PCA results, the metabolites of BF group were gradually close to AH group from 14 d to 28 d (Fig. 1A). Furthermore, PLS-DA score plot (Fig. 1B) also showed that the metabolites of the AH and BF sheep were clearly distinguishable from those of the RS group. For this reason, pathway analysis was performed for 28 d. Of the 53 metabolites, 25 important metabolites (*P* < 0.05 and VIP > 1) were identified and were used for pathway analysis based on KEGG modules (Additional file 4: Table S4). Three metabolic pathways such as glycine, serine and threonine metabolism, primary bile acid biosynthesis and biosynthesis of unsaturated fatty acids were up-regulated in BF group compared to AH group (Fig. 2A). The metabolic pathway of primary bile acid biosynthesis was up-regulated and 4 metabolic pathways were down-regulated in BF compared with RS group (Fig. 2B). Metabolic pathways involved in biosynthesis of fatty acid and bile acid were demonstrated (Fig. 2C), whereas feeding BF can improve unsaturated fatty acid metabolism via metabolisms like ko01040 (biosynthesis of unsaturated fatty acid), as well as stimulate bile acid production via metabolisms like ko00260 (glycine, serine and threonine metabolism) and ko00120 (primary bile acid biosynthesis).

### Histomorphology and gene expression of rumen epithelium

Height, width, area and thickness of rumen papillae were higher (*P* < 0.05) in AH and BF groups than in RS group (Table 4). The results of real-time PCR showed that up-regulation (*P* < 0.05) of tight junction protein-related genes such as claudin-1 and claudin-4, cell proliferation related genes such as *CDK4*, *Cyclin A2* and *Cyclin E1*, and VFA transporter-related gene such as *MCT1*, and down-regulation (*P* < 0.05) of apoptosis related genes such as caspase-8 and *Bad*, and pH regulation-related gene such as *NHE3* and *Na*^*+*^*/K*^*+*^*ATPase* were observed in BF group compared with RS group (Table 4).

**Table 4:** Effect of feeding BF on histomorphology and gene expression of rumen epithelium of sheep

Items	AH	RS	BF	SEM	*P*-value
Histomorphology of rumen epithelium					
Height of papillae, mm	3.19^a^	2.03^c^	2.70^b^	0.16	0.001
Width of papillae, mm	1.74^a^	1.20^b^	1.64^a^	0.09	0.011
Area of papillae, mm^2^	11.46^a^	5.82b^c^	9.02^ab^	0.89	0.014
Density of papillae, number/cm^2^	60.70	67.83	62.09	3.42	0.706
Total area of papillae, mm^2^/cm^2^	652.98^a^	393.87^b^	643.91^a^	45.08	0.009
Total thickness, μm	150.48^a^	126.02^b^	148.11^a^	4.09	0.008
Gene expression for nutrient transports					
*DRA*	0.42^b^	1.00^a^	1.12^a^	0.10	< 0.001
*PAT1*	0.63^b^	1.01^a^	1.02^a^	0.06	0.004
*AE2*	0.74^b^	1.02^a^	0.86^ab^	0.05	0.032
*MCT1*	2.44^a^	1.00^b^	1.78^a^	0.21	0.002
*MCT4*	1.10	1.01	1.17	0.11	0.551
*NHE1*	0.45^b^	1.00^a^	0.84^a^	0.08	< 0.001
*NHE2*	0.81	1.01	0.84	0.05	0.290
*NHE3*	0.80^b^	1.00^a^	0.78^b^	0.04	0.005
*vH*^*+*^*ATPase*	1.08	1.01	1.19	0.05	0.346
*Na*^*+*^*/K*^*+*^*ATPase*	0.67^b^	1.00^a^	0.68^b^	0.05	< 0.001
Gene expression for tight junction					
Claudin-1	0.55^c^	1.01^b^	1.22^a^	0.09	< 0.001
Claudin-4	0.90^b^	1.02^b^	1.44^a^	0.08	0.005
*ZO-1*	0.30^b^	1.00^a^	0.98^a^	0.10	< 0.001
Occludin	0.57^b^	1.02^a^	1.31^a^	0.11	0.002
Gene expression for cell proliferation					
*CDK-2*	0.86	1.02	1.09	0.05	0.219
*CDK-4*	0.92^b^	1.00^b^	1.32^a^	0.06	0.001
*CDK-6*	0.93^b^	1.01^ab^	1.22^a^	0.05	0.042
*CyclinA2*	0.56^c^	1.04^b^	1.54^a^	0.13	0.001
*CyclinD1*	0.93	1.04	0.95	0.07	0.790
*CyclinE1*	1.10^b^	1.01^b^	1.37^a^	0.06	0.007
Gene expression for cell apoptosis					
Caspase-3	0.91	1.03	0.87	0.06	0.549
Caspase-8	0.64^c^	1.01^a^	0.80^b^	0.05	0.002
*Bcl-2*	0.65^b^	1.05^ab^	1.17^a^	0.09	0.042
*Bad*	0.55^b^	1.01^a^	0.74^b^	0.06	0.002

*AH* Alfalfa hay, *RS* Rice straw, *BF* Bio-fermented rice straw

^a–c^Means within a row with different superscripts significantly different (*P* < 0.05)

### Dynamic changes of rumen fermentation parameters and microbiota

**Fig. 3:**
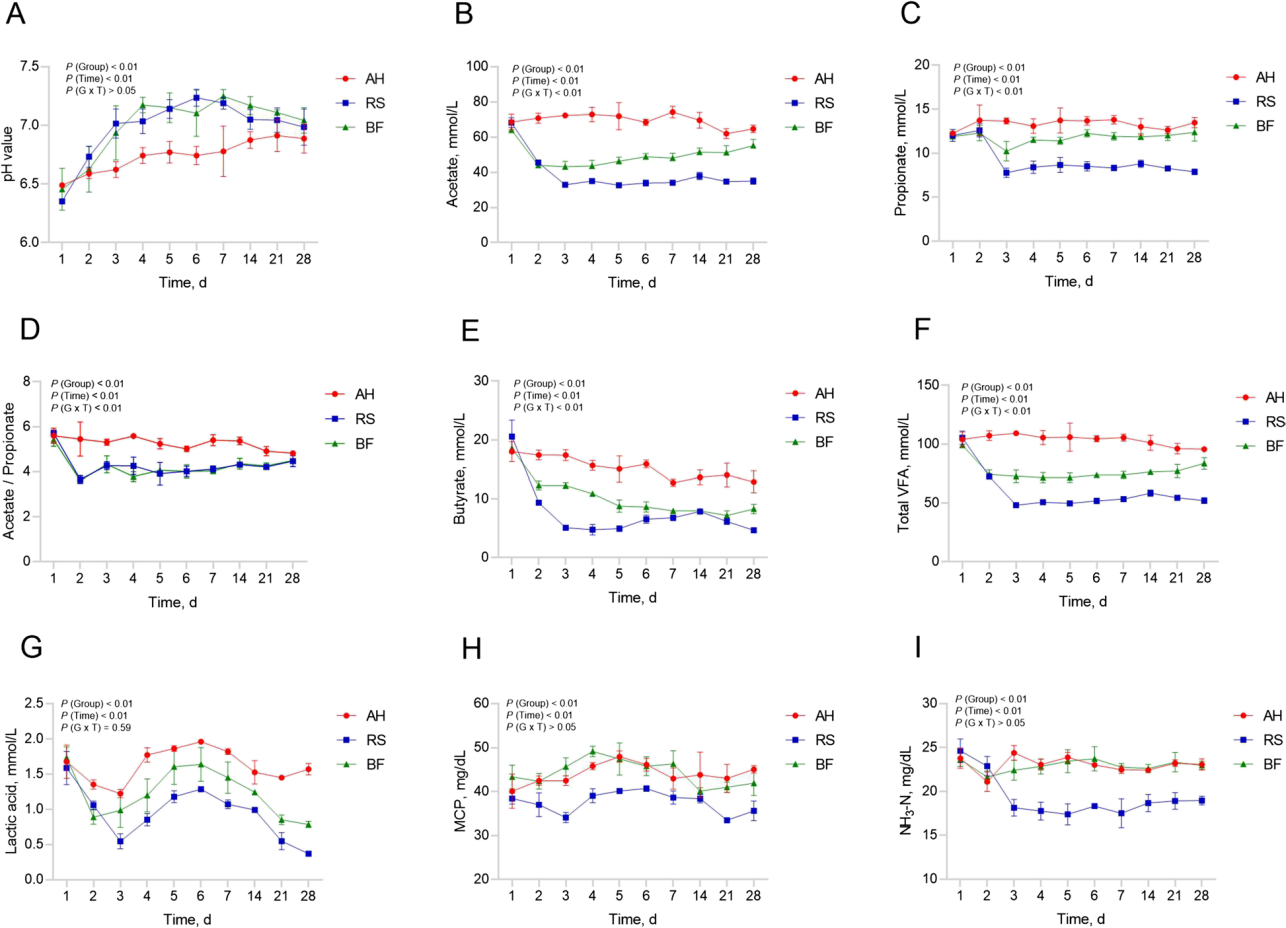
Dynamic changes of rumen fermentation parameters among AH, RS and BF groups. **A** pH; **B** Acetate; **C** Propionate; **D** Acetate/propionate; **E** Butyrate; **F** Total VFA; **G** Lactate; **H** MCP; **I** NH_3_-N

The different types of roughage and feeding time have significant effects on the dynamic changes of rumen fermentation parameters (Fig. 3). Generally, the ruminal acetate, propionate, butyrate, acetate/propionate, total VFAs and lactate were highest (*P* < 0.05) in AH group, then followed by BF and RS groups. The ruminal MCP and NH_3_-N concentrations were greater (*P* < 0.05) in AH and BF group than in RS group, while ruminal pH was lowest (*P* < 0.05) in AH group than in BF and RS groups. The most significant changes (*P* < 0.05) of ruminal fermentation parameters were observed during the first four days of experiment. These changes were afterwards gradually stabilized until the end of the experiment. For this reasons, rumen microbial community analysis was performed for the first 4 d and 28 d.

**Fig. 4:**
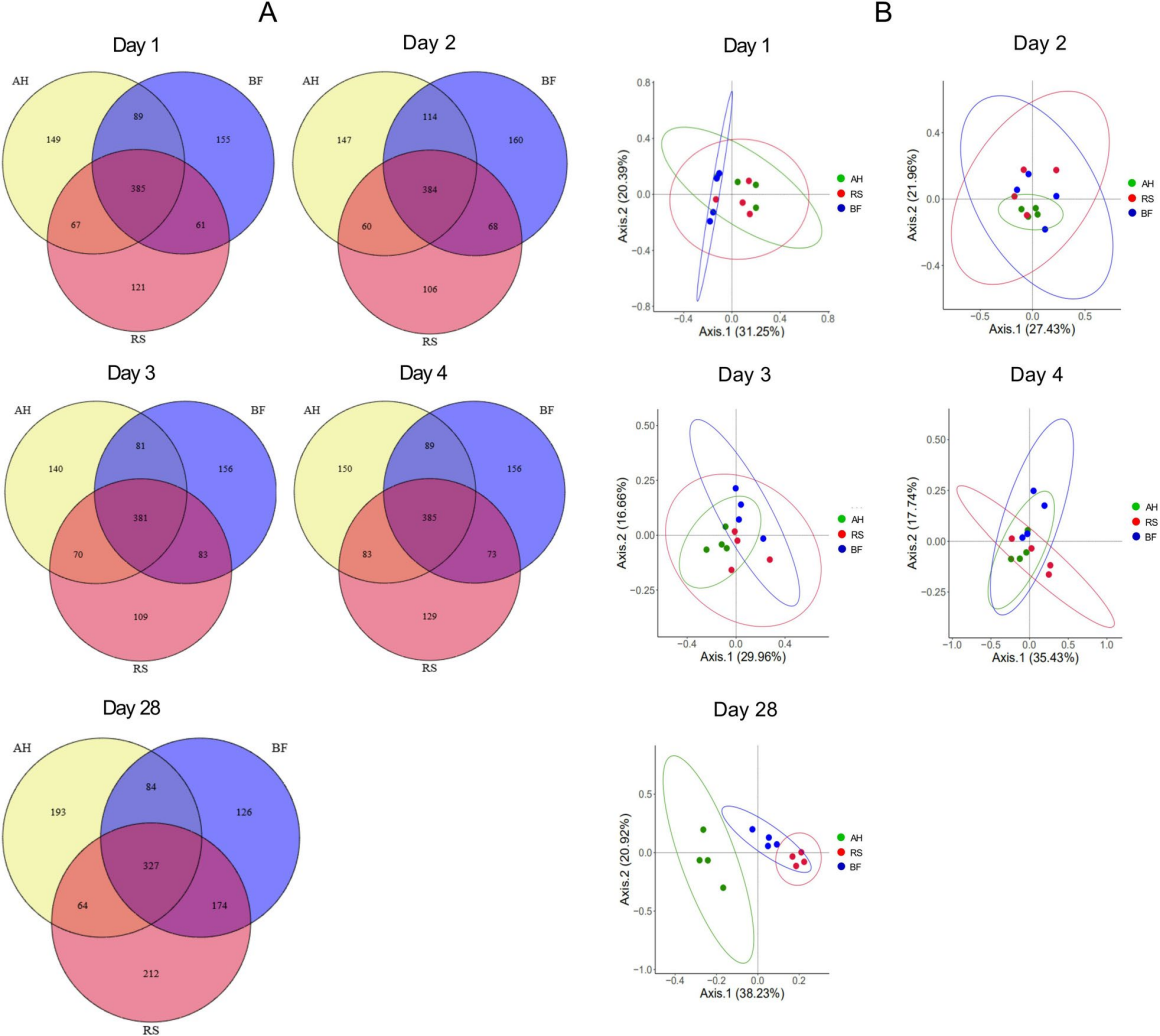
Dynamic changes of rumen microbiota among AH, RS and BF groups. **A** Venn diagrams showing the number of common and unique features; **B** Principal co-ordinates analysis (PCoA) showing the similarity or difference in the composition of rumen bacteria

The analysis of bacterial alpha diversity showed that treatment had no effect (*P* > 0.05) on the ASV, Eveness, Faith_pd, and Shannon indexes. However, time has an effect on Faith_pd, where Faith_pd on 28 d was significantly higher (*P* < 0.05) than on other days (Additional file 5: Table S5). The common and unit taxa for the first 4 d did not differ, but those for 28 d were different from those for the other days, according to Venn diagrams (Fig. 4A). For 28 d, the common taxa were 327, while the unit taxa for AH, RS and BF groups were 193, 212 and 126, respectively. The PCoA result demonstrated that the bacterial community during the first four days did not cluster separately, however it was clearly separated at the 28 d (Fig. 4B).

**Fig. 5:**
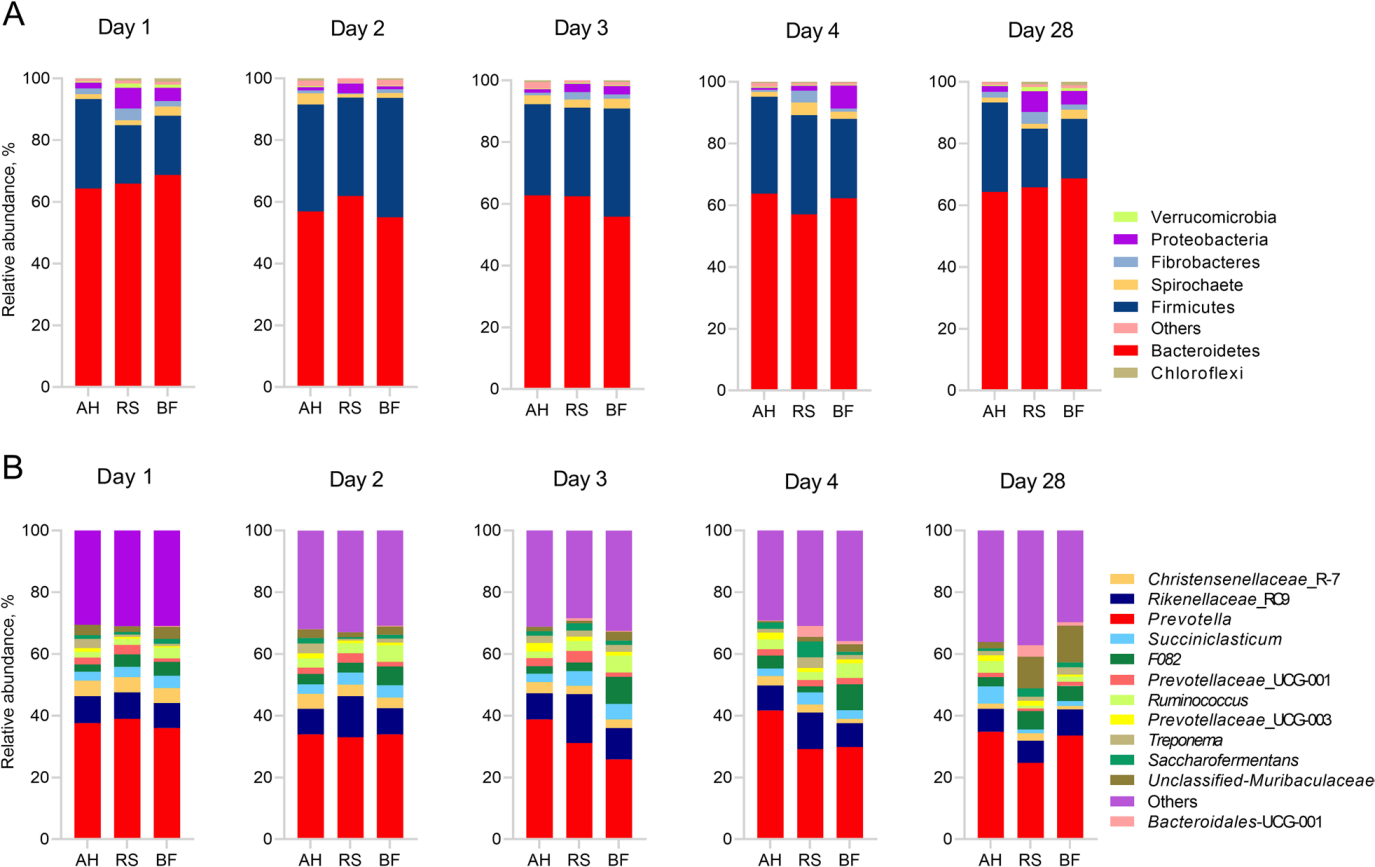
Relative abundance of rumen bacteria among AH, RS and BF groups. **A** Phylum level; **B** Genus level

Seven bacteria phyla were identified with relative abundances of more than 0.5% in at least one group, whereas Bacteroidetes and Firmicutes were most abundant, accounting for 92.45% of total bacteria for the first 4 d, and 88.66% of total bacteria for 28 d (Fig. 5A). Twelve bacteria genera were identified with relative abundances of more than 1.0% in at least one group, whereas *Prevotella* and *Rikenellaceae*_RC9 groups were most abundant, accounting for 42.81% of total bacteria for the first 4 d, and 38.72% of total bacteria for 28 d (Fig. 5B). In general, no significant influence on the relative abundance of rumen bacterial phyla and genera in sheep was found over the first 4 d, however a significant effect was observed among groups on 28 d. Therefore, LEfSe and rumen microbial KEGG modules analysis was performed for 28 d.

### Rumen microbial community analysis

**Fig. 6:**
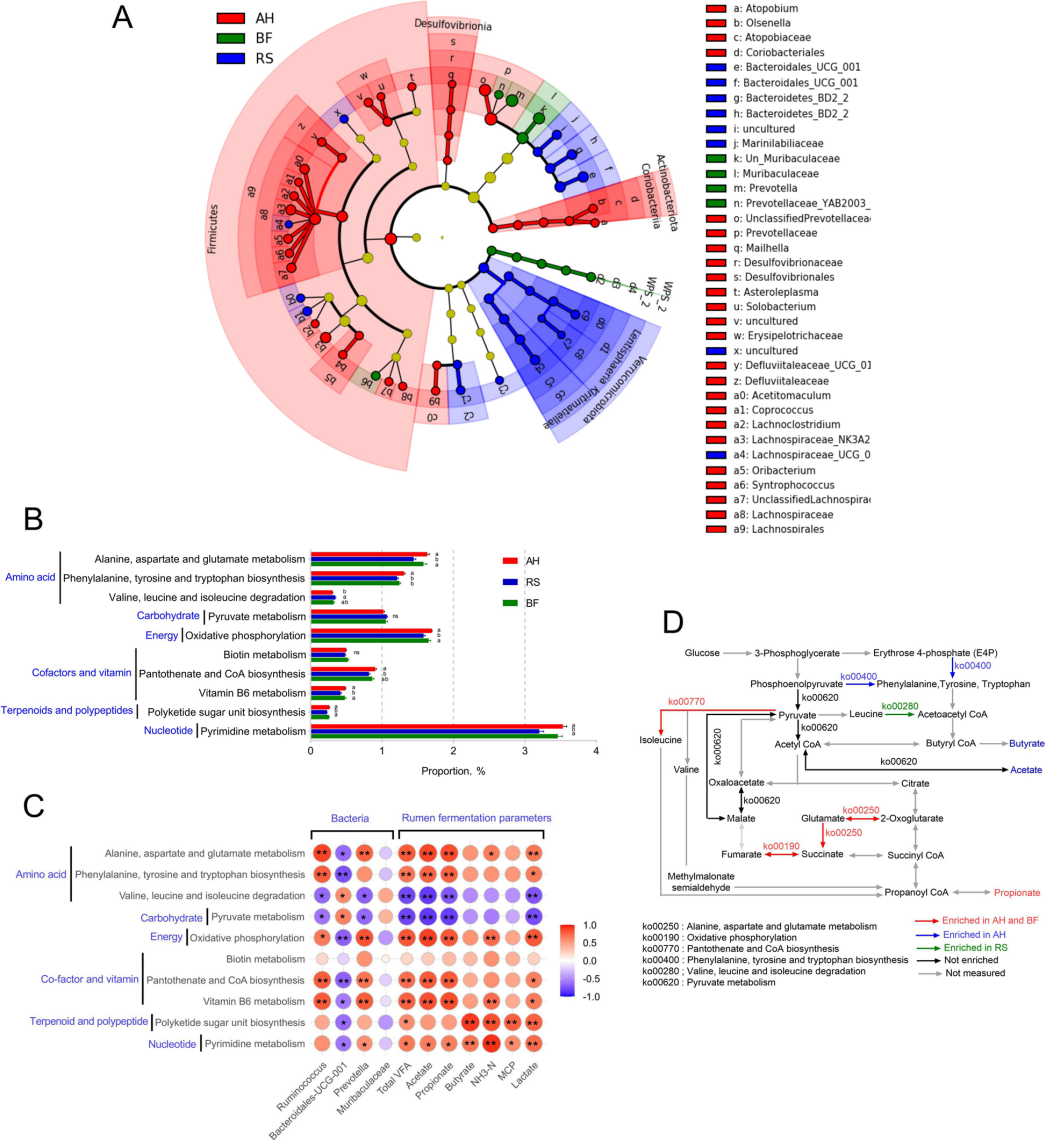
Microbial community analysis. **A** Linear discriminant analysis effect size (LEfSe) of rumen bacteria; **B** Rumen microbial KEGG modules; **C** Spearman’s correlation between rumen bacteria/rumen fermentation parameters and rumen microbial KEGG modules (^*^P < 0.05, ^**^P < 0.01); **D** Rumen bacterial KEGG modules related to biosynthesis of VFAs in the rumen of sheep

The LEfSe analysis was performed from phyla to genus level of bacteria community. Four bacterial phyla with relative abundance of > 0.5% in at least one sample and LDA score of > 2.0 were significantly different among groups, whereas Firmicutes and Actinobacteriota were higher in the AH group, Verrucomicrobia was higher in the RS group, and WPS_2 was higher in BF group (Fig. 6A). At the genus level, the 34 bacterial genera with relative abundance of > 1.0% in at least one sample and LDA score of > 2.0 were significantly different among groups, whereas 18 bacterial genera were higher in the AH group, 11 bacterial genera were higher in the RS group, and 5 bacterial genera were higher in the BF group. Then, for a better understanding, bacterial genera with LDA score of > 4.0 were analyzed separately, with *Ruminocuccus* being higher in AH group, *Bacteroidales*_UCG_001 being higher in RS group, and *Prevotella* and *un*-*Muribaculaceae* being higher in BF group (Fig. 6A).

Tax4Fun results showed that ten rumen microbial KEGG modules were enriched, whereas three modules related to amino acid metabolism, three modules related to cofactors and vitamin metabolism, one module related to carbohydrate metabolism, one related to energy metabolism, one module related to terpenoids and polypeptides, and one module related to nucleotide. Despite being higher (*P* < 0.05) than the RS group, the AH and BF groups were similar in most KEGG modules (Fig. 6B).

The Spearman’s correlation analysis was performed between rumen microbial KEGG modules and rumen bacteria (LDA score > 4 and relative abundance > 1.0%) and rumen fermentation parameters (Fig. 6C). The correlation results showed that *Ruminococcus*, *Prevotella*, total VFA, acetate, propionate and lactate were positively correlated (*P* < 0.05) with most of rumen microbial KEGG modules except valine, leucine and isoleucine degradation, and pyruvate metabolisms, which were negatively correlated (*P* < 0.05). Conversely, *Bacteroidales*_UCG_001 was negatively correlated (*P* < 0.05) with the most of rumen microbial KEGG modules. Butyrate and MCP were positively correlated (*P* < 0.05) with polyketide sugar unit biosynthesis and pyrimidine metabolism.

The involvement of rumen bacterial KEGG modules in the biosynthesis of VFAs in the rumen of sheep was constructed (Fig. 6D), whereas the modules like ko00770 (pantothenate and CoA biosynthesis), ko00250 (alanine, aspartate and glutamate metabolism) and ko00190 (oxidative phosphorylation) enriched in AH and BF groups were related to the biosynthesis of propionate, the module like ko00400 (phenylalanine, tyrosine and tryptophan biosynthesis) enriched in AH group as well as ko00280 (valine, leucine and isoleucine degradation) enriched in RS group were related to the biosynthesis of butyrate.

### *Prevotella*, propionate, methane yield, growth performances and their relationships

**Fig. 7:**
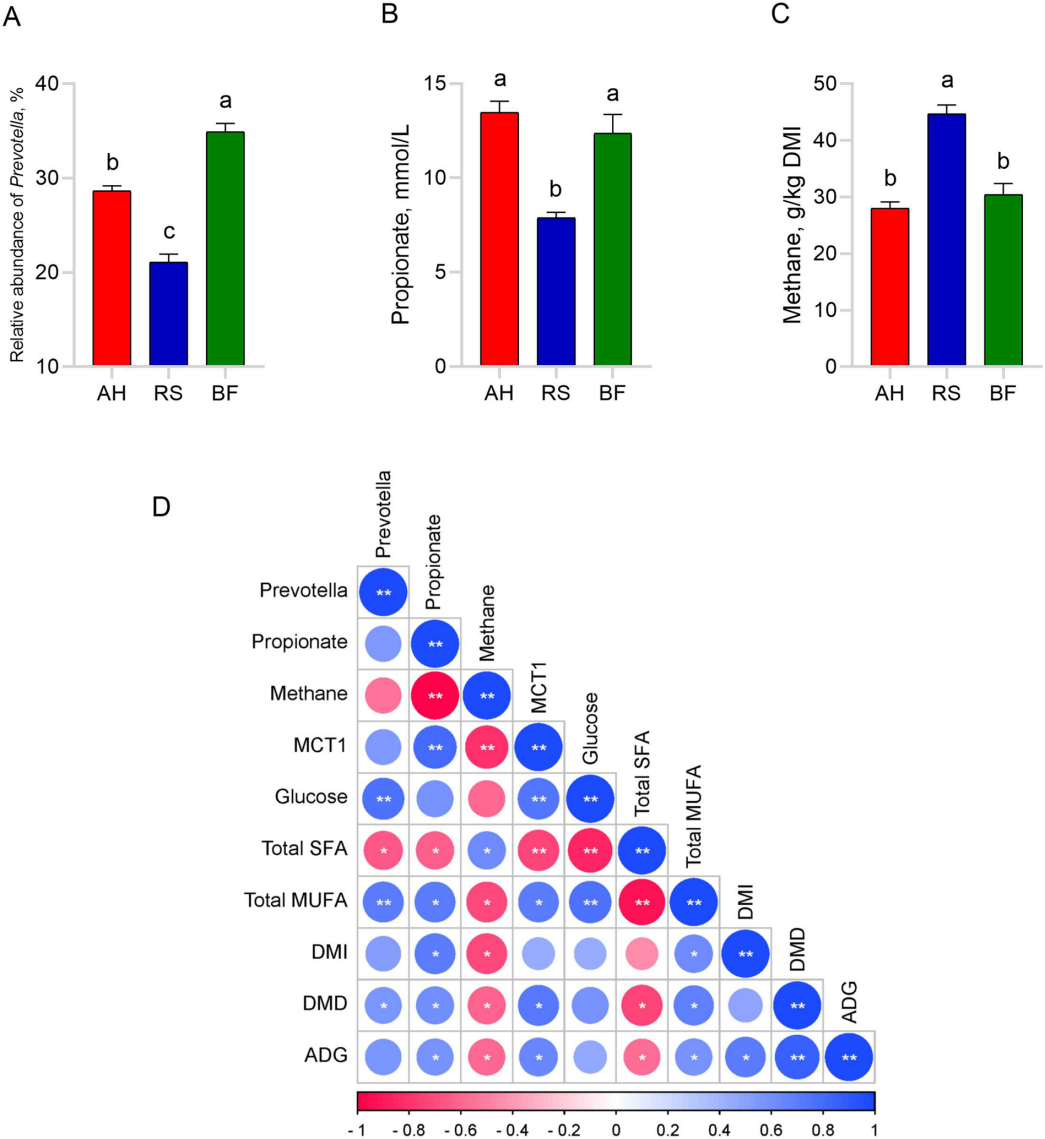
*Prevotella*, propionate and methane yield, growth performances and their relationships. **A** Relative abundance of *Prevotella*; **B** Ruminal propionate production; **C** Enteric methane yield; **D** Spearman’s correlation analysis among *Prevotella*, propionate and methane yield, growth performances (^*^P < 0.05, ^*^^*^ *P* < 0.01)

Sheep fed AH and BF had higher (*P* < 0.05) relative abundance of *Prevotella* (Fig. 7A) and ruminal propionate production (Fig. 7B), and had lower (*P* < 0.05) enteric methane yield (Fig. 7C). The Spearman’s correlation analysis was performed among the significantly different parameters of quantity and quality of performance traits (Fig. 7D). The correlation results revealed that the genus *Prevotella* and ruminal propionate content were positively correlated (*P* < 0.05) with one another. Methane was negatively correlated (*P* < 0.05) with *Prevotella*, propionate, gene expression for carbohydrate transport (*MCT1*), serum glucose concentration, total MUFA, feed intake, digestibility and growth rate of sheep, and positively correlated (*P* < 0.05) with total saturated fatty acid concentration. Except methane yield and total saturated fatty acid concentration, all other parameters were positively correlated (*P* < 0.05) with each other.

### Schematic illustration demonstrating how feeding BF to sheep improved the feed digestion, growth rate and meat quality

**Fig. 8:**
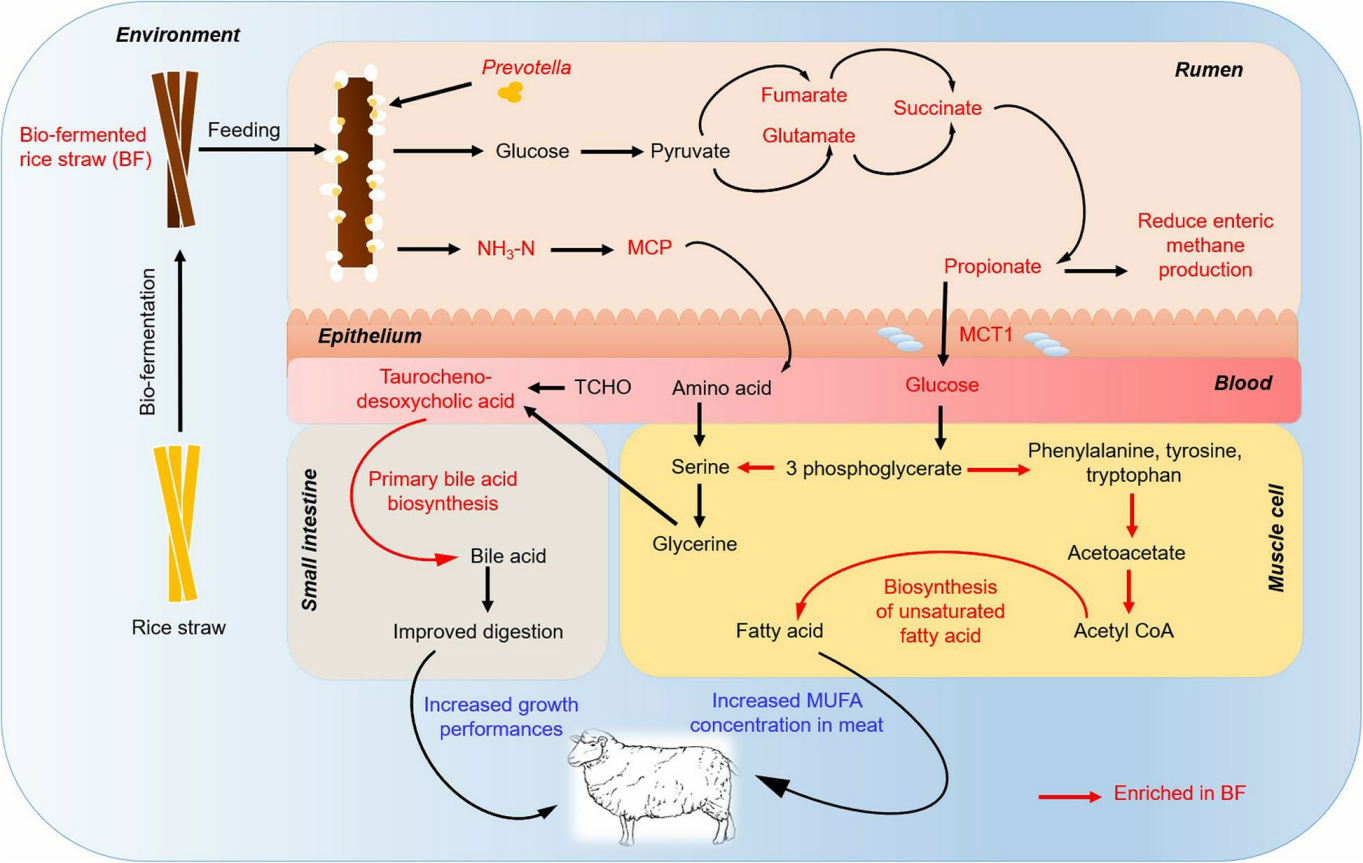
Schematic illustration demonstrating how feeding BF to sheep improved the feed digestion, growth rate and meat quality

According to the results, a schematic illustration demonstrating how feeding BF to sheep improved the feed digestion, growth rate and meat quality was created (Fig. 8). Feeding BF increased the relative abundance of *Prevotella* in rumen of sheep, which are positively related with rumen bacterial KEGG module like pantothenate and CoA biosynthesis, alanine, aspartate and glutamate metabolism and oxidative phosphorylation. In those mechanisms, pyruvate was broken down to isoleucine, glutamate was broken down to 2-oxaloglutarate and succinate, and fumarate was broken down to succinate, respectively, and lastly, they were broken down to propionate. The propionate in the rumen was transport into blood by *MCT1*, where it was transformed into glucose. Then, glucose was broken down into 3-phosphoglycerate, which were then gradually broken down into phenylalanine, tyrosine and tryptophan. Subsequently, it was broken down into acetoacetate, acetyl CoA, and lastly unsaturated fatty acid. In these breakdown processes, the metabolic pathways like phenylalanine, tyrosine and tryptophan biosynthesis, tyrosine metabolism and biosynthesis of unsaturated fatty acid were engaged. In this way, MUFA concentration was improved in meat of sheep fed BF.

Moreover, feeding BF increased the ruminal concentration of NH_3_-N and MCP, which were then broken down into serine. Additionally, 3-phosphoglycerate was also broken down into serine, which plays an essential role in several cellular processes. Serine was then broken down into taurochenodesoxycholic acid, assisting in the production of greater bile acid in the intestines, which improves feed digestion, nutrient utilization, and weight gain of sheep.

## Discussion

Improved feed intake (22%) and dry matter digestibility (11%) observed in sheep fed BF might be the direct effect of enhancement of physical characteristics and nutritional quality of RS after bio-fermentation process [19]. Greater feed digestibility and intake favored to increase the daily weight gain (53%) of sheep in BF group than RS group. Additionally, lower enteric methane yield (31%) in BF group might also support to increase the weight gain of sheep because methane is the waste of feed energy. In this manner, the energy saved from reducing methane production could be allotted to growth, leading to higher feed efficiency (31%) and lower feed cost per kg of live weight gain (20%) than RS group.

Dripping loss is the loss of fluid from meat cuts and water evaporation from the shrinkage of muscle proteins (actin and myosin) in the form of drip. Cooking loss refers to the reduction in weight of meat during the cooking process. Thus, dripping and cooking losses are of high importance due to their financial implications. High dripping loss results in an unattractive appearance as well as decreased meat tenderness and juiciness, whereas high cooking loss results in the loss of several essential minerals and vitamins, resulting in a deterioration in meat nutritional quality [34]. In this study, the lower meat dripping loss (22%) and cooking loss (28%) in muscle of sheep fed BF indicated that feeding BF might improve the meat quality and economic issue. The reasons for lowering dripping and cooking losses might be due to α-tocopherol and β-carotene in muscle [35], which were not explored in this study.

In general, saturated fat is less healthy than unsaturated fat, however both fats are important for body mechanisms. In fact, the influence of fat type on health is dependent on the ratio of unsaturated fat to saturated fat, with a higher ratio being more healthful than a lower ratio [36]. The optimum ΣMUFA/ΣSFA ratio is ranging from 0.8 to around 0.95. In this study, lower ΣSFA (12%) and, higher ΣMUFA (18%) and ΣMUFA/ΣSFA ratio (35%) were observed in the muscle of sheep in FB group, indicating that feeding FB improved the healthy fat content in the meat of sheep. The primary factors influencing the fatty acid composition of meat are the age of the animal, breed type, and feed [37]. The age and breed type of the animals in this investigation were comparable. As the feed factor, the chemical compositions of the experimental feed were not different, however the sources of forages used were different, which could explain differences in fatty acid synthesis in the body. Feeding BF upregulated the biosynthesis of unsaturated fatty acid, which resulted in higher unsaturated fatty acid concentrations in the BF group.

Feeding BF improved the serum AST level, which reflected liver protein metabolism, playing a role in amino acid metabolism and in the urea and tricarboxylic acid cycles [38]. The concentrations of serum total protein, albumin and glucose were increased in BF group because bio-fermentation increased the contents of ruminal NH_3_-N and fermentation carbohydrate of RS [20]. As the results of pathway analysis, the metabolic pathways related to the glycine, serine, and threonine metabolism, primary bile acid biosynthesis, and the biosynthesis of unsaturated fatty acids, were up-regulated in the BF group. Higher serum glucose and betaine concentrations in the BF group may be associated with the up-regulation of glycine, serine, and threonine metabolism. Glycine is generated from serine, which is derived from 3-phosphoglycerate, an intermediate of glycolysis. Furthermore, betaine is one of the sources of glycine formation [39]. Higher taurochenodesoxycholic acid concentrations in the BF group may be a key component in the up-regulation of primary bile acid biosynthesis. Primary bile acids, like cholic acid, chenodeoxycholic acid, and taurochenodesoxycholic acid, are steroid carboxylic acids generated from cholesterol in vertebrates. These primary bile acids are conjugated with glycine for the secretion of bile into the intestine [40]. As a result of increased bile acid secretion into the intestines, nutrient digestion and absorption were enhanced in the BF group, leading to higher growth performances. Fatty acids are normally synthesized from acetyl-CoA, which is derived from glucose via pyruvate. Additionally, oleic acid is also one of major component for the biosynthesis of unsaturated fatty acid [41]. Thus, increasing serum glucose and oleic acid concentration in BF group could associated with the up-regulating biosynthesis of unsaturated fatty acid, resulting the higher MUFA in the meat of BF group.

Feeding BF to sheep significantly increased the height, width, and unit area of the rumen papillae, which may be attributed to an increase in nutrient transport via ruminal wall. Related researches [42, 43] had demonstrated that the rumen epithelium absorbs and transports about 50%–80% of VFA in the rumen, and the effectiveness of the transport is positively correlated with the surface area of the epithelium and the expression of transporters.

There are 3 different types of carriers used in the transport of VFA by the rumen epithelium; VFA^–^/H^+^ exchange carrier (DRA, PAT1, AE2), VFA^–^/H^+^ co-transporter (MCT1, MCT4) and cell homeostatic regulatory proteins (NHE-1, NHE-2, NHE-3, VH^+^ATP, Na^+^/K^+^ATP). The VFA^−^/H^+^ exchange carrier transports HCO_3_^–^ to the outside of the cell while transporting VFA^–^ into the cell; the VFA^–^/H^+^ co-transporter can transport VFA, lactic acid and other substances into the blood to provide energy for the body; and the cell homeostasis regulatory protein is mainly responsible for equal transport of excess H^+^ in the cell and Na^+^ outside the cell to prevent cytoplasmic acidification [44]. As a result, the up-regulation of *MCT1* and the down-regulation of *NHE*-*3* and *Na*^+^/*K*^+^*ATP* genes in BF group demonstrated that feeding BF triggered the expression of VFA^–^/H^+^ co-transporter without involving cell homeostatic regulatory proteins. Up-regulation of claudin-1 and claudin-4 in BF group suggested that feeding BF was beneficial to the health of sheep by optimizing rumen epithelial barrier function. The expression of the claudin-4 gene was markedly down-regulated in goats with damaged rumen epithelial barrier function [45]. Up-regulation of genes related to cell proliferation (*CDK*-*4*, *CyclinA2*, *CyclinE1*) and down-regulation of genes related to cell apoptosis (caspase-8, *Bad*) observed in BF group is supported by the report, where feeding high-grain diet could promote the development of rumen epithelium, which is achieved by enhancing the cell proliferation and inhibiting the cell apoptosis of rumen epithelium [42].

The sudden changes of feed offered at the start of formal trial resulted the most significant changes of rumen fermentation parameters over the first four days of the experiment. The researcher [46] stated that rumen bacterial community changes in first week after rapid changes of diet. These changes were then gradually stabilized until the experiment completed. This indicated that the rumen microbial community had become gradually stable after four days of sudden feed changes, resulting in gradually steady rumen fermentation parameters until the experiment's completion. Feeding BF improved the ruminal VFAs of sheep compared with feeding RS, which might be due to the greater availability of fermentable carbohydrate. Bio-fermentation increased the fermentable carbohydrate content of RS and up-regulated energy metabolism [19]. Although all experimental diets were isonitrogenously formulated, NH_3_-N and MCP were higher in the BF group than the RS group, which might be due to the increased protein breakdown into ammonia by bio-fermentation and higher MCP synthesis from ammonia. Therefore, it could be assumed that feeding BF increased protein metabolism and energy supply for growth of animal.

Treatment has no effect on the bacterial alpha diversity; however, time has an effect on Faith_pd. This might be due to the interactive effect between feed offered and duration of experiment. The researcher [47] stated that the structural composition of the rumen bacterial community can be affected by a great number of internal and external factors, such as host, physiological status, diet, and environment.

Bacteroidetes and Firmicutes were the most dominant bacterial phyla in this study, which was supported by the report [48], stated that the most predominant bacterial phyla in the goat’s rumen are Bacteroidetes and Firmicutes. Those two bacterial phyla are associated with the fiber and polysaccharide degradation and are considered to be the primary degrader of complex soluble polysaccharides in plant cell walls [49]. At the genus level, *Prevotella* and *Rikenellaceae*_RC9 gut groups were the most predominant bacterial genera, which is consistent with the finding [50], claimed that these two bacterial genera were most abundant bacterial genera in the rumen of sheep. *Prevotella* has a great functional versatility and is mainly involved in carbohydrate and nitrogen metabolisms in the rumen, as well as in producing a variety of enzymes involved in the degradation of starch, proteins, peptides, and hemicellulose [51, 52]. Propionate synthesis by *Prevotella* species is important for maintaining glucose homeostasis in host animals through gluconeogenesis [51]. The *Rikenellaceae*_ RC9 gut group is associated with primary or secondary carbohydrate degradation and protein fermentation [53]. Therefore, the dynamic changes in ruminal microbial community could help in understanding how forage and rumen microbes interact [16] and could be manipulated to increase the energy supply within the rumen and improve feed energy efficiency and weight gain [54].

According to the LEfSe analysis, the genus *Ruminocuccus*, which is involved in cellulose degradation and produce large amounts of cellulase [52], was significantly higher in AH group, resulting greater feed digestion and efficiency in that group. *Bacteroidales*_UCG_001, which was higher in RS group, is more abundant in high-forage than in low-forage diets and it has been associated with fiber digestion [47] and biohydrogenation of fatty acids in the rumen [55]. Therefore, lower feed digestibility, feed efficiency and unsaturated fatty acid composition, and higher saturated fatty acid composition were found in RS group. *Prevotella*, which was higher in the BF group, is primarily involved in carbohydrate and nitrogen metabolism in the rumen and provides enzymes for hemicellulose degradation [52]. Thus, feed digestion and efficiency were improved in BF group.

According to Tax4Fun analysis, the bacterial KEGG module related to the biosynthesis of VFAs such as ko00770 (pantothenate and CoA biosynthesis), ko00190 (oxidative phosphorylation), ko00250 (alanine, aspartate, and glutamate metabolism) and ko00400 (phenylalanine, tyrosine and tryptophan biosynthesis) were enriched in AH and BF group, while ko00280 (valine, leucine and isoleucine degradation) was enriched in RS group. These KEGG modules were positively correlated with *Ruminococcus*, *Prevotella* and fermentation products, and negatively correlated with *Bacteroidales*_UCG_001. Pantothenate and CoA biosynthesis is linked to valine/isoleucine biosynthesis, which involves the breakdown of pyruvate into valine and isoleucine. Through propenol CoA, they were subsequently transformed to propionate [56]. Oxidative phosphorylation is a type of energy metabolism that involves the breakdown of fumarate into succinate, which is then converted to succinyl CoA [57]. Alanine, aspartate, and glutamate metabolism is a kind of amino acid metabolism in which glutamate is broken down into 2-oxaloglutarate and succinate, which are then converted to succinyl CoA [58]. The succinyl CoA was transformed into propionate via the propanoyl CoA. Phenylalanine, tyrosine and tryptophan biosynthesis is also a type of amino acid metabolism, in which phenylalanine, tyrosine and tryptophan were generated from the phosphoenolpyruvate and erythrose 4-phosphate (E4P), and then converted into acetoacetyl CoA [59]. In valine, leucine and isoleucine degradation, especially leucine degradation, leucine is ultimately converted into acetoacetyl CoA [60], which was broken down into butyrate via butyryl CoA.

As mentioned above, increased production of VFAs, especially propionate (56%), was due to the greater availability of fermentable carbohydrate after bio-fermentation of RS [19]. Correlation analysis also revealed that *Prevotella* was positively correlated with propionate content and negatively related with enteric methane yield. The researchers stated that *Prevotella* can increase propionate concentration and limit methanogenesis [61], and increasing the population of *Prevotella* could reduce methane production [62]. Furthermore, the propionate formation competes with methanogenesis for metabolic hydrogen utilization in rumen and could reduce the enteric methane emission [63]. This supports our findings that feeding BF to sheep reduced the enteric methane yield (31%). Thus, the reduction of enteric methane production might be significantly influenced by *Prevotella*, which requires more investigation in the forthcoming research. *Prevotella* was found to be positively correlated to total MUFA and negatively related to total SFA [64], which supports our findings. Moreover, feed digestion and weight gain were also associated with that bacterium, which might be due to their greater fiber degrading efficiency [52] and their ability in reducing enteric methane production, thereby minimizing feed energy waste and optimizing growth rate of sheep. Production performances such as MUFA, feed intake, digestibility and weight gain were associated with rumen fermentation parameters and *MCT1*. After degradation of feed consumed by *Prevotella*, the VFAs were produced in the rumen, which facilitated to enhance the carbohydrate transportation like *MCT1* gene expression. Increased carbohydrate transportation, especially propionate, leads to enhance serum glucose level, which is also positively related with production performances.

## Conclusion

Feeding BF changed the rumen microbial community, particularly increasing the relative abundance of *Prevotella*, which improved the ruminal propionate production, reduced enteric methane yield and enhanced carbohydrate transport into the blood. Additionally, changes in serum metabolome up-regulated the primary bile acid biosynthesis and biosynthesis of unsaturated fatty acid, resulting improved feed intake, digestion, growth rate and meat quality. Consequently, improving the feed efficiency and lowering the feed cost per kg of live weight were achieved. Therefore, bio-fermentation of rice straw could be an innovative way for improving ruminant production with minimizing production costs.

### Supplementary Information

**Additional file 1: Table S1.** Chemical compositions of feedstuffs.

**Additional file 2: Table S2.** Primers sequences used for quantitative real-time PCR analysis.

**Additional file 3: Table S3.** Effect of feeding BF on serum biochemical parameters of sheep.

**Additional file 4: Table S4.** Effect of feeding BF on serum metabolites of sheep.

**Additional file 5: Table S5.** Effects of feeding BF on rumen bacteria alpha diversity of sheep.

## Data Availability

The data analyzed during the current study are available from the corresponding author on reasonable request.

## Abbreviations

ADF: Acid detergent fiber
ADL: Acid detergent lignin
AH: Alfalfa hay
ALB: Albumin
ALP: Alkaline phosphatase
ALT: Alanine aminotransferase
AST: Aspartate aminotransferase
BF: Bio-fermented rice straw
DM: Dry matter
DMI: Dry matter intake
FC: fold change
GLOB: Globulin
GLU: Glucose
HDL: High-density lipoprotein
IMF: Intramuscular fat
LAB: Lactic acid bacteria
MCP: Microbial protein
ME: Metabolizable energy
MUFA: Mono-unsaturated fatty acid
NDF: Neutral detergent fiber
NH_3_-N: Ammonia nitrogen
OM: Organic matter
PUFA: Poly-unsaturated fatty acid
RS: Rice straw
SFA: Saturated fatty acid
TCHO: Total cholesterol
TP: Total protein
TRIG: Triglycerides
VFA: Volatile fatty acid
VIP: Variable importance in projection
