# Supplementary material for: Dynamic changes of rumen microbiota and serum metabolome revealed increases in meat quality and growth performances of sheep fed bio-fermented rice straw
Source: J Anim Sci Biotechnol. 2024 Feb 28;15:34. doi: 10.1186/s40104-023-00983-5 (PMC10900626; doi:10.1186/s40104-023-00983-5)
Supplement: Supplementary file 1 — Additional file 1: Table S1. Chemical compositions of feedstuffs. [file 40104_2023_983_MOESM1_ESM.docx]

**Additional file 1**

**Table S1** Chemical compositions of feedstuffs

| **Items** | **Roughage** | | |  | **Concentrate mixture** | |
| --- | --- | --- | --- | --- | --- | --- |
|  | **AH** | **RS** | **BF** |  | **CMAH** | **CMRS** |
| DE, kcal/kg | 2,374 | 2,068 | 2,109 |  | 3,217 | 3,471 |
| ME, kcal/kg | 2,397 | 2,088 | 2,130 |  | 3,249 | 3,505 |
| DM, % | 83.92 | 82.37 | 46.19 |  | 82.73 | 82.29 |
| CP, % | 19.45 | 4.76 | 4.99 |  | 8.37 | 17.63 |
| EE, % | 3.54 | 2.98 | 2.75 |  | 2.08 | 1.83 |
| Ash, % | 13.48 | 14.15 | 14.19 |  | 11.01 | 7.74 |
| NDF, % | 46.27 | 72.15 | 66.97 |  | 13.57 | 8.06 |
| ADF, % | 20.54 | 32.23 | 31.65 |  | 6.58 | 4.32 |
| ADL, % | 3.11 | 4.59 | 3.40 |  | 0.00 | 0.00 |

AH: Alfalfa hay; RS: Rice straw; BF: Bio-fermented rice straw; DE: Digestible energy; ME: Metabolizable energy; DM: Dry matter; CP: Crude protein; EE: Ether extract; NDF: Neutral detergent fiber; ADF: Acid detergent fiber; ADL: Acid detergent lignin; CMAH: Concentrate mixture for AH group including corn (50.00%), wheat middling (40.83%), zeolite (4.17%), premix (4.17%), and NaCO₃ (0.83%); CMRS: Concentrate mixture for RS and BF groups including corn (55.00%), bean pulp (30.83%), wheat middling (8.33%), limestone (0.83%), premix (4.17%), and NaCO₃ (0.83%)
