# Supplementary material for: Dynamic changes of rumen microbiota and serum metabolome revealed increases in meat quality and growth performances of sheep fed bio-fermented rice straw
Source: J Anim Sci Biotechnol. 2024 Feb 28;15:34. doi: 10.1186/s40104-023-00983-5 (PMC10900626; doi:10.1186/s40104-023-00983-5)
Supplement: Supplementary file 2 — Additional file 2: Table S2. Primers sequences used for quantitative real-time PCR analysis. [file 40104_2023_983_MOESM2_ESM.docx]

**Additional file 2**

**Table S2** Primers sequences used for quantitative real-time PCR analysis

| **Gene name** | **Primer sequence (5’→3’)** | **Gene ID/reference** | **Amplicon size, bp** |
| --- | --- | --- | --- |
| *Claudin-1* | F: CACCCTTGGCATGAAGTGTA | [44] | 216 |
|  | R: AGCCAATGAAGAGAGCCTGA |  |  |
| *Claudin-4* | F: AAGGTGTACGACTCGCTGCT | [44] | 238 |
|  | R: GACGTTGTTAGCCGTCCAG |  |  |
| *ZO-1* | F: CGACCAGATCCTCAGGGTAA | [44] | 163 |
|  | R: AATCACCCACATCGGATTCT |  |  |
| *Occludin* | F: GTTCGACCAATGCTCTCTCAG | [44] | 200 |
|  | R: CAGCTCCCATTAAGGTTCCA |  |  |
| *GAPDH* | F: GGGTCATCATCTCTGCACCT | NM_001190390.1 | 180 |
|  | R: GGTCATAAGTCCCTCCACGA |  |  |
| *DRA* | F: CCTAAAATCAACCTCCACA | NW_014639013.1 | 145 |
|  | R: TCATCATCAGTTCCAGCAA |  |  |
| *PAT-1* | F: TGGACTGCCCCCCGTGTAT | NW_011942300.1 | 391 |
|  | R: CCAGAGCGGCTGCTTAGAT |  |  |
| *AE2* | F: AGCAGCAACAACCTGGAGT | [63] | 155 |
|  | R: GGTGAAACGGGAGACGAA |  |  |
| *MCT-1* | F: ATCTACGCGGGATTCTTTGGAT | [63] | 72 |
|  | R: AAGGTCCATCAGCGTTTCAAAC |  |  |
| *MCT-4* | F: GTTTGGGATAGGCTACAGTGACACA | [63] | 107 |
|  | R: GCAGCCAAAGCGATTCACA |  |  |
| *vH^+^ ATPase* | F: TTTTATTGAACAAGAAGCCAATGA | [65] | 143 |
|  | R: GATTCATCAAATTGGACATCTGAA |  |  |
| *Na^+^/K^+^ ATPase* | F: TGAGCATCCCAGTGTTGT | [65] | 180 |
|  | R: CCTTGTCCAGATACTTCCT |  |  |
| *NHE-1* | F: GAAGAAAAAGCAAGAAACG | NW_014639011.1 | 218 |
|  | R: TTGTGGTAGAAAGCGATGA |  |  |
| *NHE-2* | F: GAGACATAGCAAAGGATAC | NW_014639012.1 | 122 |
|  | R: TAGAAGAAAGGGACAAGAG |  |  |
| *NHE-3* | F: GTGCTCCTGACGCTACTTT | NW_011942323.1 | 378 |
|  | R: TGTCCGATTTGCCCTGATA |  |  |
| *CDK2* | F: CCTAGCTTTCTGCCACTCTCAT | NM_001142509.1 | 153 |
|  | R: TCACCACCTCGTGGGTATAAGT |  |  |
| *CDK4* | F: GACCAAGACCTCAGGACGTATC | NM_001127269.1 | 250 |
|  | R: CACCACTTGTCACCAGAATGTT |  |  |
| *CDK6* | F: GATGGCTCTTACCTCAGTGGTT | XM_012177413.2 | 228 |
|  | R: GGGTAGGGCAACATCTCTAGG |  |  |
| *Cyclin A2* | F: CTCTCCTATCACCGCCTGAC | NC_019478.2 | 142 |
|  | R: CTTTGGGGTCCAAGTTCTGC |  |  |
| *Cyclin D1* | F: CCTGCCGTCCATGCGGAA | XM_015102997.1 | 103 |
|  | R: GAACTTCACATCTGTGGCAC |  |  |
| *Cyclin E1* | F: TGGCACCGATGTCTCTGTTC | XM_015100542.1 | 114 |
|  | R: CCACACTGGCTTCTCACAGT |  |  |
| *Caspase-3* | F: CAGCTACCTCAAACACAGTTGG | XM_015104559.1 | 203 |
|  | R: TGATACAGTGGCATACCCACAT |  |  |
| *Caspase-8* | F: TCCAGGATTCGCCTCTGGTA | NC_019459.2 | 133 |
|  | R: CCGGCTTAGGAACTTGAGGG |  |  |
| *Bcl-2* | F: GTGGATGACCGAGTACCTGAAC | XM_012103831.2 | 197 |
|  | R: CTTCACTTATGGCCCAGATAGG |  |  |
| *Bad* | F: TTTCGGAAGACTGAGGTCTGAT | XM_004019650.3 | 185 |
|  | R: CGGCGAAGTTAGGGTTAATCTC |  |  |
