# Supplementary material for: Dynamic changes of rumen microbiota and serum metabolome revealed increases in meat quality and growth performances of sheep fed bio-fermented rice straw
Source: J Anim Sci Biotechnol. 2024 Feb 28;15:34. doi: 10.1186/s40104-023-00983-5 (PMC10900626; doi:10.1186/s40104-023-00983-5)
Supplement: Supplementary file 3 — Additional file 3: Table S3. Effect of feeding BF on serum biochemical parameters of sheep. [file 40104_2023_983_MOESM3_ESM.docx]

**Additional file 3**

**Table S3** Effect of feeding BF on serum biochemical parameters of sheep

| **Items** | | **AST, U/L** | **ALT, U/L** | **ALP, U/L** | **TP,**  **g/L** | **ALB, g/L** | **GLOB, g/L** | **A/G** | **UREA, mmol/L** | **GLU, mmol/L** | **TRIG, mmol/L** | **TCHO, mmol/L** | **HDL, mmol/L** |
| --- | --- | --- | --- | --- | --- | --- | --- | --- | --- | --- | --- | --- | --- |
| Groups | |  |  |  |  |  |  |  |  |  |  |  |  |
|  | AH | 76.65^a^ | 11.75 | 314.05 | 80.92^a^ | 26.77^ab^ | 54.16 | 0.52 | 6.79 | 3.27^a^ | 0.24 | 1.15 | 0.63 |
|  | RS | 62.63^b^ | 10.87 | 302.10 | 74.58^b^ | 26.19^b^ | 48.40 | 0.54 | 7.19 | 2.81^b^ | 0.24 | 1.07 | 0.61 |
|  | BF | 72.32^a^ | 10.80 | 285.20 | 80.15^a^ | 27.45^a^ | 52.69 | 0.53 | 7.69 | 3.30^a^ | 0.23 | 1.17 | 0.64 |
| Time | |  |  |  |  |  |  |  |  |  |  |  |  |
|  | Day 1 | 87.83^a^ | 11.42 | 246.25^b^ | 80.62 | 25.94^b^ | 54.69 | 0.49 | 5.92^b^ | 3.24 | 0.21 | 1.12 | 0.61 |
|  | Day 7 | 64.83^bc^ | 12.11 | 304.67^ab^ | 79.98 | 26.40^b^ | 53.58 | 0.51 | 7.13^b^ | 3.06 | 0.25 | 1.05 | 0.6 |
|  | Day 14 | 67.92^bc^ | 10.83 | 297.00^ab^ | 77.15 | 26.65^b^ | 50.51 | 0.52 | 7.47^a^ | 3.1 | 0.23 | 1.12 | 0.65 |
|  | Day 21 | 60.00^c^ | 10.42 | 307.50^ab^ | 77.36 | 26.38^b^ | 50.96 | 0.53 | 7.66^a^ | 3.15 | 0.25 | 1.1 | 0.62 |
|  | Day 28 | 72.08^b^ | 10.92 | 346.83^a^ | 77.63 | 28.63^a^ | 49.00 | 0.58 | 7.94^a^ | 3.09 | 0.24 | 1.26 | 0.65 |
| SEM | | 3.29 | 0.74 | 21.06 | 1.99 | 0.46 | 2.24 | 0.03 | 0.45 | 0.08 | 0.02 | 0.09 | 0.03 |
| *P*-value | |  |  |  |  |  |  |  |  |  |  |  |  |
|  | Diet (D) | 0.001 | 0.431 | 0.46 | 0.01 | 0.05 | 0.061 | 0.694 | 0.197 | 0.000 | 0.774 | 0.584 | 0.538 |
|  | Time (T) | 0.000 | 0.549 | 0.032 | 0.617 | 0.001 | 0.376 | 0.137 | 0.026 | 0.485 | 0.569 | 0.546 | 0.685 |
|  | D × T | 0.794 | 0.845 | 0.654 | 0.932 | 0.201 | 0.920 | 0.867 | 0.177 | 0.246 | 0.600 | 0.569 | 0.985 |

AH: Alfalfa hay; RS: Rice straw; BF: Bio-fermented rice straw; AST: Aspartate aminotransferase; ALT: Alanine aminotransferase; ALP: Alkaline phosphatase; TP: Total protein; ALB: Albumin; GLOB: Globulin; A/G: Albumin/globulin; GLU: Glucose; TRIG: Triglycerides; TCHO: Total cholesterol; HDL: High-density lipoprotein

^a–c^Means within a column with different superscripts significantly different (*P* < 0.05)
