# Supplementary material for: Dynamic changes of rumen microbiota and serum metabolome revealed increases in meat quality and growth performances of sheep fed bio-fermented rice straw
Source: J Anim Sci Biotechnol. 2024 Feb 28;15:34. doi: 10.1186/s40104-023-00983-5 (PMC10900626; doi:10.1186/s40104-023-00983-5)
Supplement: Supplementary file 4 — Additional file 4: Table S4. Effect of feeding BF on serum metabolites of sheep. [file 40104_2023_983_MOESM4_ESM.docx]

**Additional file 4**

**Table S4** Effect of feeding BF on serum metabolites of sheep

| **Classification** | **Metabolites** | **Groups** | | |  | **FC** | | ***P*-value** | **VIP** |
| --- | --- | --- | --- | --- | --- | --- | --- | --- | --- |
|  |  | **AH** | **RS** | **BF** |  | **BF/AH** | **BF/RS** |  |  |
| Lipid | 12,13-DHOME | 8,514^a^ | 5,313^b^ | 8,456^a^ |  | 0.99 | 1.59 | 0.023 | 1.09 |
|  | 1-Linoleoylglycerophosphocholine | 1,661^a^ | 1,160^b^ | 1,314^b^ |  | 0.79 | 1.13 | 0.002 | 1.15 |
|  | 9,10-DiHODE | 5,586^a^ | 3,692^b^ | 3,463^b^ |  | 0.62 | 0.94 | 0.041 | 1.17 |
|  | LysoPC (16:0) | 2,524^a^ | 1,932^b^ | 2,626^a^ |  | 1.04 | 1.36 | 0.030 | 1.11 |
|  | LysoPC (18:0/0:0) | 2,519^a^ | 1,738^b^ | 2,376^a^ |  | 0.94 | 1.37 | 0.002 | 1.16 |
|  | LysoPE (0:0/14:0) | 27,775^a^ | 3,842^b^ | 25,526^a^ |  | 0.92 | 6.64 | 0.000 | 1.36 |
|  | LysoPE (18:0/0:0) | 3549^a^ | 2,465^b^ | 3,549^a^ |  | 1.00 | 1.44 | 0.048 | 1.01 |
| Fatty acid | 10Z-Heptadecenoic acid | 7,126^a^ | 3,397^b^ | 6,545^a^ |  | 0.92 | 1.93 | 0.001 | 1.22 |
|  | 11,12-Epoxyeicosatrienoic acid | 4,289^a^ | 2,110^b^ | 4,257^a^ |  | 0.99 | 2.02 | 0.002 | 1.24 |
|  | Eicosapentaenoic acid | 3829^a^ | 1744^b^ | 2,320^b^ |  | 0.61 | 1.33 | 0.001 | 1.21 |
|  | Linoleic acid | 24,834^a^ | 14,160^b^ | 21,935^a^ |  | 0.88 | 1.55 | 0.002 | 1.13 |
|  | Oleic Acid | 44,793^b^ | 43,089^b^ | 62,992^a^ |  | 1.41 | 1.46 | 0.001 | 1.66 |
|  | Stearic acid | 2,594^a^ | 2,264^ab^ | 1,650^b^ |  | 0.64 | 0.73 | 0.044 | 1.32 |
|  | α-Linolenic acid | 6,272^a^ | 2,061^b^ | 3,497^b^ |  | 0.56 | 1.70 | 0.002 | 1.15 |
| Organic acid | 2-Hydroxy-2-methylbutyric acid | 16,166^a^ | 10,103^b^ | 15,599^a^ |  | 0.96 | 1.54 | 0.002 | 1.20 |
|  | Ricinoleic acid | 7,588^a^ | 5,077^b^ | 7,776^a^ |  | 1.02 | 1.53 | 0.011 | 1.17 |
| Amino acid | Betaine | 16,5042^b^ | 236,544^a^ | 211,707^a^ |  | 1.28 | 0.89 | 0.013 | 1.03 |
|  | Citrulline | 15,771^a^ | 11,441^b^ | 11,546^b^ |  | 0.73 | 1.01 | 0.020 | 1.12 |
|  | DL-2-Aminooctanoic acid | 5,867^b^ | 5,449^b^ | 10,497^a^ |  | 1.79 | 1.93 | 0.004 | 1.60 |
|  | L-Tryptophan | 2,887^a^ | 1,826^b^ | 2,166^b^ |  | 0.75 | 1.19 | 0.014 | 1.04 |
| Alkaloids | Hypoxanthine | 5,452^a^ | 2,964^c^ | 4,039^b^ |  | 0.74 | 1.36 | 0.000 | 1.19 |
| Indoles | Indoleacrylic acid | 3,646^a^ | 2,298^c^ | 3,178a |  | 0.87 | 1.38 | 0.005 | 1.10 |
| Choline | Acetylcholine | 4,596^a^ | 3,316^b^ | 3,251^b^ |  | 0.71 | 0.98 | 0.018 | 1.17 |
| Pyridines | 4-Pyridoxic acid | 1,776^a^ | 818^c^ | 1,227^b^ |  | 0.69 | 1.50 | 0.001 | 1.18 |
| Steroid | Taurochenodesoxycholic acid | 12,001^b^ | 3,987^c^ | 28,148^a^ |  | 2.35 | 7.06 | 0.000 | 1.74 |

AH: Alfalfa hay; RS: Rice straw; BF: Bio-fermented rice straw; FC: fold change; VIP: Variable importance in projection

^a–c^Means within a row with different superscripts significantly different (*P* < 0.05)
