# Supplementary material for: Dynamic changes of rumen microbiota and serum metabolome revealed increases in meat quality and growth performances of sheep fed bio-fermented rice straw
Source: J Anim Sci Biotechnol. 2024 Feb 28;15:34. doi: 10.1186/s40104-023-00983-5 (PMC10900626; doi:10.1186/s40104-023-00983-5)
Supplement: Supplementary file 5 — Additional file 5: Table S5. Effects of feeding BF on rumen bacteria alpha diversity of sheep. [file 40104_2023_983_MOESM5_ESM.docx]

**Additional file 5**

**Table S5** Effects of Feeding BF on rumen bacteria alpha diversity of sheep

| **Items** | **Groups** | | |  | **Times** | | | | | **SEM** | ***P*-value** | | |
| --- | --- | --- | --- | --- | --- | --- | --- | --- | --- | --- | --- | --- | --- |
|  | **AH** | **RS** | **BF** |  | **Day 1** | **Day 2** | **Day 3** | **Day 4** | **Day 28** |  | **Group** | **Time** | **G × T** |
| ASV | 349.10 | 344.15 | 359.55 |  | 337.67 | 344.67 | 346.25 | 344.42 | 381.67 | 25.92 | 0.634 | 0.260 | 0.633 |
| Evenness | 0.70 | 0.71 | 0.72 |  | 0.71 | 0.73 | 0.72 | 0.70 | 0.68 | 0.02 | 0.332 | 0.143 | 0.483 |
| Faith_pd | 26.66 | 26.83 | 27.74 |  | 25.62^b^ | 26.07^b^ | 26.54^b^ | 27.71^ab^ | 29.44^a^ | 1.40 | 0.426 | 0.012 | 0.194 |
| Shannon | 5.88 | 6.00 | 6.08 |  | 5.96 | 6.10 | 6.10 | 5.91 | 5.86 | 0.24 | 0.417 | 0.618 | 0.431 |

AH: Alfalfa hay; RS: Rice straw; BF: Bio-fermented rice straw; ASV: amplicon sequence variant

^a,b^Means within a row with different superscripts significantly different (*P* < 0.05)
